# Phytochemicals: A Promising Weapon in the Arsenal against Antibiotic-Resistant Bacteria

**DOI:** 10.3390/antibiotics10091044

**Published:** 2021-08-26

**Authors:** Bahman Khameneh, N. A. Michael Eskin, Milad Iranshahy, Bibi Sedigheh Fazly Bazzaz

**Affiliations:** 1 Department of Pharmaceutical Control, School of Pharmacy, Mashhad University of Medical Sciences, Mashhad 9177948954, Iran; khamenehbagherib@mums.ac.ir; 2 Department of Food and Human Nutritional Sciences, Faculty of Agricultural and Food Sciences, University of Manitoba, Winnipeg, MB R3T 2N2, Canada; michael.eskin@umanitoba.ca; 3 Department of Pharmacognosy, School of Pharmacy, Mashhad University of Medical Sciences, Mashhad 9177948954, Iran; 4 Biotechnology Research Center, Pharmaceutical Technology Institute, Mashhad University of Medical Sciences, Mashhad 9177948954, Iran

**Keywords:** phytochemicals, antibiotic resistance, complementary medicine, herbal medicine, clinical applications

## Abstract

The extensive usage of antibiotics and the rapid emergence of antimicrobial-resistant microbes (AMR) are becoming important global public health issues. Many solutions to these problems have been proposed, including developing alternative compounds with antimicrobial activities, managing existing antimicrobials, and rapidly detecting AMR pathogens. Among all of them, employing alternative compounds such as phytochemicals alone or in combination with other antibacterial agents appears to be both an effective and safe strategy for battling against these pathogens. The present review summarizes the scientific evidence on the biochemical, pharmacological, and clinical aspects of phytochemicals used to treat microbial pathogenesis. A wide range of commercial products are currently available on the market. Their well-documented clinical efficacy suggests that phytomedicines are valuable sources of new types of antimicrobial agents for future use. Innovative approaches and methodologies for identifying plant-derived products effective against AMR are also proposed in this review.

## 1. Introduction

According to various surveys, there is a direct relationship between the increased use of antibiotics and the creation of resistant bacteria. The appearance of resistant microorganisms to drugs leads to the currently available treatment regimes becoming less effective or totally ineffective [1,2,3,4]. As a result, this has become a prominent issue and a serious concern for global health agencies such as the World Health Organization, Centers for Disease Control, and regional health ministries. Additionally, it represents a challenging problem for the medical fraternity [5,6]. In addition, the effectiveness of antibiotics has been substantially reduced by the existence of different resistance mechanisms. Of these, the major reasons are antibiotic inactivation by enzyme production, alteration of drug targets, changes in cell permeability, the intrinsic expression of efflux pumps, and biofilm formation. The last one, in particular, acts as a defense against drugs and contributes to the sustained persistence of resistant bacteria.

The first antibiotic, penicillin, was discovered by Alexander Fleming in 1928. Since then, other β-lactam antibiotics identified include cephalosporins, carbapenems, and monobactams. They all contain a four-membered cyclic ring consisting of three carbon atoms and one nitrogen atom. The nitrogen atom is attached to the β-carbon relative to the carbonyl group, and hence the name β-lactam is used.

Fleming [7] reported that several bacteria in the colityphoid group were not inhibited by penicillin. Subsequent work by Abraham and Chain [8] identified an enzyme in Gram-negative *Escherichia coli*, penicillinase, capable of destroying penicillin. This enzyme, now referred to as β-lactamase, is responsible for destroying the β-lactam ring in penicillin and other β-lactam antibiotics. Initially, very few bacteria produced β-lactamase, but the overuse of antibiotics has resulted in its widespread production by Gram-negative bacteria rendering them resistant to many life-saving antibiotics. Among the many strategies being investigated to overcome these resistant bacteria is to inhibit the β-lactamase enzyme. Synthetic compounds have been developed for inhibiting β-lactamase. However, concerns regarding their toxicity have resulted in a concerted effort to find safer plant sources of inhibitors.

In addition, mobile genetic elements such as plasmids, insertion sequences, transposons, and integrative conjugative elements all play an important role in developing resistance against antibacterial agents [4].

Under these circumstances and to overcome this problem, identifying alternative or complementary approaches is urgently needed to prevent and treat microbial infections. One such approach employs naturally occurring compounds with potential antibacterial activities [3,9,10,11,12,13]. Herbal medicines are rich in various compounds, such as alkaloids, flavonoids, terpenoids, coumarins, tannins, antimicrobial peptides, and steroids, which can be used as an alternative or complement to conventional antibiotics [14,15,16,17,18]. These compounds exert their antimicrobial activities via different mechanisms, including (I) structural disruption of the bacterial cell and increase in cell permeability and leakage of cell constituents, (II) alterations in the bacterial cell wall and cell membrane, (III) losing ATP, (IV) inhibition of protein synthesis, (V) intracytoplasmic damage, pH disturbance, DNA damage and (VI) inhibition of quorum sensing among bacteria [10,19,20,21]. Plant-derived compounds are generally less expensive, safer to use in terms of side effects, and more readily available than their synthetic counterparts [21,22]. Therefore, the isolation and characterization of plant-derived substances with suitable antibacterial activities are integral for developing natural antibacterial agents.

Despite a significant increase in publications on this topic, mechanisms of action and their clinical use have remained elusive. This review describes different aspects of phytochemicals, such as identification, characterization, and evaluation of their biological activities. In the next step, we will explain the valuable findings of preclinical and clinical studies of some phytomedicine in the market worldwide.

We conducted a systematic review and searched different databases such as PubMed, Embase, Scopus, Web of Science, and Google Scholar without time limitations.

## 2. Isolation, Characterization, and Bioassays of Phytochemicals

As mentioned earlier, using naturally occurring compounds with potential antibacterial activities has been considered an alternative or complementary in treating infectious diseases. Therefore, identifying bioactive components and understanding their properties play a vital role in evaluating phytochemicals [23,24]. Conventional screening methods, including disk diffusion, TLC-direct bioautography, and broth microdilution antibiotic sensitivity test, have been used extensively to screen the antimicrobial activities of extracts and purely natural compounds. These methods have helped discover almost every antibiotic available in the market and the identification of drug susceptibility. Despite the advantages of well-established procedures, classical methods suffer from several limitations such as unsatisfactory test speed, high cost, and low reliability.

Moreover, these methods provide no information about the mechanism of action of the crude extract or pure natural product that leads to the rediscovery of the compounds with a similar mechanism of action. Thus, several studies tried to offer some solutions to overcome some of the drawbacks mentioned above. This section will discuss the recent advanced development of the methods used for antimicrobial screening of phytochemicals against antibiotic-resistant microorganisms.

### 2.1. Microfluidic Technology

Establishing a high-throughput screening platform with high resolution and speed has attracted natural products to discover antibacterial drugs. Although droplet-based microfluidic technology is nascent, it continues to show promise in many biomedical fields and has revolutionized our understanding of single-cell interactions [25].

Recent studies confirmed the capability of microfluidic technology to rapidly and precisely screen novel antibiotic candidates, specifically against antibacterial-resistant mutants.

Dhayakaran et al. [25] developed a 3D microfluidic device to assess the antibacterial activity of synthesized soy peptides PGTAVFK and IKAF-KEATKVDKVVVLWTA against *Pseudomonas aeruginosa* and *Listeria monocytogenes*. The bottom layer was a glass layer; 24 incubation chambers were in the middle polydimethylsiloxane layer, and in the final layer were concentration generating gradients. Using the device, the authors were able to determine the antimicrobial activity of the peptides based on their optical density at 600 nm easily without any need for prior serial dilution. These devices can be successfully employed to screen huge libraries of natural products or crude extracts and substantially reduce the time needed for high-throughput screening. While a considerable effort has been spent establishing microfluidic technology to screen natural products against cancer [26], there are very few studies on the antibacterial drug screening of natural products using this technology. Future studies are desperately needed in this field. 

### 2.2. Host-Pathogen Co-Culture Assay

Co-culturing of human cell lines and pathogenic bacteria in the presence of a natural antimicrobial product or crude extract can simultaneously determine the efficacy and tolerability of the antibacterial candidates. Human cell lines are incubated with an antibacterial candidate and then infected with the desired pathogenic bacteria in this method. In this way, the minimal inhibitory concentration or dose and the selectivity index are determined precisely. This is beneficial for optimization and high-throughput screening because of time- and cost-efficiencies [27].

In this regard, Haque et al. [28] evaluated the antimicrobial activity of semisynthetic derivatives of betulin as a triterpenoid natural product. Two derivatives showed promising activity against Gram-positive bacteria in broth microdilution assays. However, in the host-pathogen co-culture assay, weak or no activity was observed for derivatives. Further studies in the presence of an increased albumin concentration showed that betulin derivatives could potently bind to albumin present in human cell line culture media. Hence, the host-pathogen co-culture assay can predict a drug candidate’s serum protein binding potential and guides the researcher through therapeutic potential and pharmacokinetics in the primary steps of antibiotic drug discovery from natural products.

### 2.3. Colorimetric Assay of pH

A high-throughput screening method that is sensitive and robust gives more detailed information than the minimum bactericidal concentration (MBC) and minimum inhibitory concentration (MIC) and is highly desirable for finding natural products with a novel mechanism of action. Such a method was proposed by Ymele-Lek et al. [29], who used thymol blue and bromothymol blue as pH-sensitive dyes. As fermentation can decrease the pH of the culture medium, using these dyes coupled with the colorimetric assay enabled the authors to screen 39,000 crude extracts and find suitable candidates to inhibit bacterial sugar fermentation. This simple colorimetric assay led to the identification of a broad-spectrum antimicrobial natural product, mirandamycin. Further studies revealed that mirandamycin is active against *E. coli*, *P. aeruginosa*, methicillin-resistant *Staphylococcus aureus* (MRSA), and *Mycobacterium tuberculosis*.

These studies indicate that the development of novel screening methods has the same importance and impact as screening novel sources of natural products on antibacterial drug discovery.

### 2.4. In Silico Screening

With over 300,000 entries in SciFinder that have never been tested for their antibacterial activity, natural products are valuable sources of compounds that can be harnessed for discovering future antibacterial drugs [30]. However, the rapid exploitation of such potential with conventional methods of screening seems impossible. Using the advantages of in silico, however, one can successfully screen a plethora of entries in a database to identify chemical structures that can inhibit critical enzymes in bacteria in a matter of a few days. This method has several advantages, including ease of access and saving time and money. However, there are some limitations, i.e., these screenings can only be performed on proteins and enzymes whose 3D protein crystal structure is available in the appropriate databases [31].

An example of such a successful approach was recently published by Skariyachan and co-workers [32]. They filtered 236 natural compounds from the Super Natural II database for the ADMET properties using the PreADMET and SwissADME websites. Six lead compounds were selected for further docking studies in the binding site of proteins that are significantly involved in antibiotic resistance in *Acinetobacter baumannii*, including orotate phosphoribosyltransferase (PyrE), Orotidine 5′-phosphate decarboxylase (PyrF), outer membrane protein 38 (Omp38), and Protein RecAm. Among the natural drug ligands, 16-epiestriol exhibited the best binding potential to all proteins, especially PyrE, with a −7.3 kcal/mol binding energy. In vitro studies revealed that 16- epiestriol at 200 µg/mL could significantly inhibit clinically isolated multidrug-resistant *A. baumannii* [32]. 16-Epiestriol is considered a lead compound, and by chemical optimization, more potent compounds can be synthesized in the future to overcome the antibacterial resistance in *A. baumannii*. This study demonstrates that a rational design of in silico screening studies may be valuable in the possible post-antibiotic era.

## 3. Mechanistic Insights on Phytochemicals

**Figure 1:**
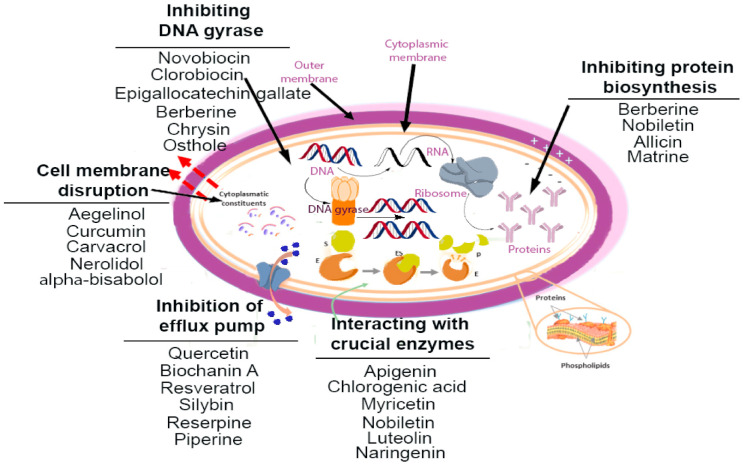
Antibacterial mechanism of action of plant-derivative compounds.

Phytochemicals show promising results in overcoming the resistance development of resistance in bacterial pathogens and combating bacterial infections. These compounds can restore the clinical application of conventional antibiotics by increasing their potency and avoiding the development of resistance. The antibacterial properties of phytochemicals are related to chemically interference with the function or synthesis of vital components and circumventing the mechanism of antibacterial resistance. Different mechanisms have been mentioned in antibacterial actions that inhibit bacterial cell-wall biosynthesis and cell membrane destruction, inhibiting bacterial protein biosynthesis, DNA replication and repairing, and metabolic pathways [33,34]. In addition, different mechanisms are involved in bacterial resistance to an antibiotic, such as overexpression of the efflux pumps, destroying the antibacterial agents, structural modification of porins, and modification of antibiotics [33,35]. Therefore, inhibition of them is an integral part of combating antibiotic resistance [3]. Phytochemicals based on their chemical structures and properties could exhibit antibacterial actions via one or more of these mechanisms [4]. Based on structures, they are categorized into major groups of alkaloids, tannins, carbohydrates, and glycosides, terpenoids, flavonoids, steroids, and coumarins [36]. These compounds have particular clinical value because their bioactivity generally does not lead to resistance. Some important plant-derivative compounds with antibacterial activities and their mechanism of action are illustrated in Figure 1.

Polyphenols show antibacterial activity against a broad spectrum of bacteria. Among them, flavanols, flavonols, and phenolic acids exhibit the highest activities because of (I) inhibiting bacterial virulence factors including enzymes and toxins, (II) interacting with the cytoplasmic membrane or reducing the pH values, (III) suppressing biofilm formation, (IV) exerting synergistic effects with conventional antibiotics, and (V) reducing the extracellular polysaccharide (EP) activity and acting as EP inhibitors (EPIs) [37,38,39]. Phenolic compounds that are produced in relatively high concentrations show promising EPI activity against pathogenic bacteria. They could inhibit cell wall biosynthesis and critical enzymes such as urease, sortase A and dihydrofolate reductase. The Fabaceae family has the most phenolic derivative compounds among botanical families [35].

It should be noted that their activity is mainly weak and also non-specific. However, in some cases, target specificity among phytochemicals has been reported. For example, coumarins have high activity against *S. aureus,* while no activity against Gram-negative bacteria has been observed [40].

Zhao et al. [41] extracted, purified, and identified a specific β-lactamase inhibitor in green tea, epigallocatechin gallate (EGCG). They tested it on 21 clinical isolates of penicillinase-producing *S. aureus*. In addition to direct binding with peptidoglycan in the bacterial cell membrane, ECGG exhibited a dose-dependent inhibition of penicillinase activity with a 50% inhibition at a concentration of 10 µg/ml. A later study by Zhao et al. [42] reported restoring the antibacterial activity of β-lactams (cefotaxime and imipenem) in the presence of EGCG against a series of β-lactamase producing bacteria, including 21 *S. aureus*, 6 *E. coli*, 3 *Klebsiella pneumonia*, and 8 *Serratia marcescens* strains. The in vitro studies showed the effectiveness of ECGG in β-lactamase activity inhibition and restoring the antibacterial activities of penicillin. However, in vivo studies exhibited less effectiveness due to the intracellular location of the enzymes and the protective permeability barrier of the cell walls and cell membranes [43].

An interesting flavonoid, galangin, was reported in the rhizomes of the perennial plant *Alpinia officinarum* by Eukeb et al. [44]. Galangin was effective in reversing the β-lactam antibiotic resistance of *S. aureus*. This led Sirlwong and co-workers [45] to examine the synergism between several other flavonoids, quercetin or kaempferol, in combination with amoxicillin for their ability to overcome amoxicillin-resistant *Staphylococcus epidermis* (ARSE). The synergy between quercetin and amoxicillin proved to be very effective by inhibiting peptidoglycan synthesis in the bacterial cell membrane, inhibiting β-lactamase activity, increasing cell membrane permeability, and increasing protein amide I and II, and decreasing fatty acids in the bacterial cells. The authors pointed out the need to determine their safety and efficacy using animal and human subjects.

The increased resistance of *Streptococcus* spp. to antibiotics is one of the significant causes of mastitis. This condition is an inflammation of the mammary gland that results in major economic losses to the dairy industry [46]. To combat this problem, Maia and co-workers [47] isolated guttiferone-A and 7-epiclusianone from the fruits of *Garcinia brasiliensis*, a tree native to the Amazon and widely cultivated in Brazil. The pharmacological properties of these two bioactive compounds, particularly their antimicrobial properties against *S. aureus* and *Bacillus cereus* [48], suggested their possible prevention of metastasis. Synergistic effects were evident between 7-epiclusianone and guttiferone-A with ampicillin or gentamicin. At levels below their MIC values, both compounds reversed the antibiotic resistance of *Streptococcus agalactine* and *Streptococcus uberis*. Neither compounds were cytotoxic, and their strong binding of β-lactamase could explain the reversal of ampicillin resistance. Their potential for the treatment of bovine mastitis appears promising.

A root canal infection such as apical periodontitis is a severe problem worldwide. The infection is primarily caused by the growth of *Prevotella* spp., *Porphyromonas* spp., *Fusobacterium* spp., *Enterpcococcus* spp., and *Candida* spp. While chemical irritants are used to eliminate these multidrug-resistant organisms, they generally fail, with the residual organisms causing tissue necrosis, gastritis, and local inflammation. To overcome this problem, Sriramkumar et al. [49] undertook the homology modeling of the β-lactamase protein from *Staphylococcus sciuri* and docking studies with 4-butanylanisole and 9-ocatadecene. These phytochemicals were extracted from *Garcinia imberti*, a flowering plant of the family Clusiaceae growing in India. Both compounds exhibited favorable inhibitory activity of β-lactamase by binding with the conserved amino acids glutamine, asparagine, lysine, and phenylalanine at their active site. Based on this information, antimicrobial compounds can be tailored for a specific organism, such as *S. sciuri*.

Resveratrol is another phenolic compound with potential antibacterial properties. It is active against multidrug-resistant (MDR) Gram-negative bacteria with MICs ranging from 32 μg/mL to 128 μg/mL. The possible mechanism of action is related to the inhibition of the efflux pump activities [50,51].

Sophoraflavanone G is another potent antibacterial agent. This compound can inhibit the growth of MRSA via different mechanisms such as interacting with peptidoglycan and inhibiting cell wall biosynthesis [52,53].

Baicalein is an effective bactericide. The results of the study indicated that this compound had pronounced antibacterial activities on S. aureus. The mechanism could affect bacterial membrane penetrability, inhibit protein synthesis, and influence SDH, MDH, and DNA topoisomerase I and II activities to exert the antibacterial functions [54].

Quercetin and luteolin are other phenolic compounds with promising antibacterial activities. These compounds can increase cytoplasmic membrane permeability, caused irregular cell shape, peptidoglycan, and CM damage, and decrease nucleic acid content but increase proteins in bacterial cells. Luteolin and quercetin propose the potential to develop adjuncts to conventional antibiotics to treat infectious diseases [55].

Alkaloids are another important group of compounds that possess antibacterial properties. They are heterocyclic nitrogen compounds with highly variable chemical structures. Their antibacterial activities have been proven, and many studies have reported that they can play a significant role in treating infectious diseases. Their mechanism of action might be due to the enzymatic alterations affecting physiological processes, including inhibition of DNA synthesis and repair mechanisms by intercalating nucleic acids [9,56,57]. Isoquinolines, aporphines, quinolones, and phenanthrenes are the most critical alkaloid groups with suitable antibacterial activities [35].

It was shown that berberine had certain inhibitory effects on four common bacteria with MICs for E. coli, B. subtilis, S. aureus, and Salmonella were 2.40, 3.60, 3.30, and 3.95 mg/mL, respectively. Scanning electron microscopy showed that berberine damaged the morphology of the bacterial cells and ruptured the cells, leading to the leakage of intracellular sub-stances. Consequently, the nucleic acid content in the bacterial suspension was increased remarkably. The polyacrylamide gel electrophoresis analysis indicated that berberine could inhibit protein synthesis. Additionally, this compound could reduce the Na+/K+-ATPase activity of the cell mem-brane. Therefore, berberine inhibited the expression of bacterial proteins by destroying the cell membrane structures, which finally leads to the death of the cells so that it can exert good antibacterial effects and can be used as a valuable antibacterial agent [58].

Reserpine as an inhibitor of efflux pumps was shown to reduce the resistance of MRSA strains to conventional antibiotics [59].

Sanguinarine, a benzophenanthridin alkaloid, strongly induced fila-mentation in Gram-positive and -negative bacteria and prevented bacterial cell division by inhibiting cytokinesis. Sanguinarine inhibited bacterial division by perturbing FtsZ assembly dynamics in the Z ring. These observations support the hypothesis that the assembly and bundling of FtsZ play an important role in bacterial growth cytokinesis. Thus sanguinarine may be used as a lead compound to develop FtsZ-targeted antibacterial agents [60].

The investigations of the competitive binding of antibiotics and caffeine with DNA show that at physiological concentrations of antibiotic and caffeine (mM), the dominant mechanism influencing the affinity of the antibiotic with DNA is the displacement of bound antibiotic molecules from DNA due to caffeine-DNA complexation. These observations explain the protector actions of caffeine [61]. Sulfur-containing compounds are another critical group of phytochemicals with antibacterial and antifungal activities. They have exerted antibacterial activities against both Gram-positive and -negative bacteria. In addition, it has been shown that plants with high concentrations exert a broad spectrum of antimicrobial activities [62].

Allicin exhibited promising antifungal activities against different pathogens. The putative mechanisms of action are influencing DNA replication, mitochondrial translation, and chromatids cohesion. These pro-cesses play a critical role in yeast cells’ cell cycle, growth, and viability [63].

Isothiocyanates derived from cruciferous plants reveal antibacterial activity. They showed antibacterial activities against E. coli, K. pneumonia, S. aureus, S. epidermidis, B. subtilis, and E. faecalis. E. coli strains. They are effective against different pathogenic bacteria and act by at least two mechanisms depending on bacteria species. These compounds exert their antibacterial effects by acting on cell membranes and leakage of cellular metabolites [64,65].

The coumarins are heterocyclic compounds found in various plants. They exert a wide range of bioactive properties such as anticoagulant, antibacterial, antiviral, antioxidant, anti-inflammatory, antitumor, and enzyme inhibition. The antibacterial activity of coumarins is mainly due to inhibiting bacterial DNA gyrase, preventing supercoiling [66].

The dichloromethane extract of Prangos hulusii has yielded nine known and one new prenylated coumarins. The root extract and its prenylated coumarins exhibit antibacterial activities against nine stand-ards and six clinically isolated strains at concentrations between 5 and 125 µg/mL [67].

Aegelinol and agasyllin showed antibacterial activities against nine ATCC and the same clinically isolated Gram-positive and -negative bacterial strains. At a concentration between 16 and 125 mg/mL, both coumarins showed remarkable antibacterial effects against Gram-negative and -positive bacteria [68].

Terpenes or isoprenoids are widely outspread in nature, have high biological activity. They show a broad spectrum of antibacterial activities via different mechanisms. Their mechanism is closely associated with their lipophilic features. Monoterpenes preferentially could impact the membrane structures and increase the fluidity and permeability, altering the topology of its proteins and making disturbances across the respiration chain [69]. In addition, they could change the membrane permeability without cell lysis. 

Carvacrol is a monoterpenic phenol, biosynthesized from γ-terpinene through p-cymene. This compound occurs in aromatic plants and many essential oils of the Labiatae family. Carvacrol is reported to have a wide range of biological properties, including antibacterial activities. Compared to other volatile compounds present in essential oils, the compound shows higher antibacterial power because of the phenol ring, which confers hydrophobicity and also the presence of the free hydroxyl group. Carvacrol is active against many Gram-positive and -negative human pathogenic bacteria. In particular, it is very effective in controlling foodborne pathogens, such as E. coli, Salmonella, and B. cereus [70,71].

Thymol is a carvacrol isomer also known as “hydroxy cymene”. The compound possesses antibacterial activities against a wide range of species, including biofilm-embedded microorganisms [72].

**Table 1:** The most prevalent natural antibacterial compounds with the related mechanism of action.

Compound	Chemical Structure	Active against (MIC/MBC Values)	Class of Phytochemicals	Mechanism of Action	Ref.
**Piperine**	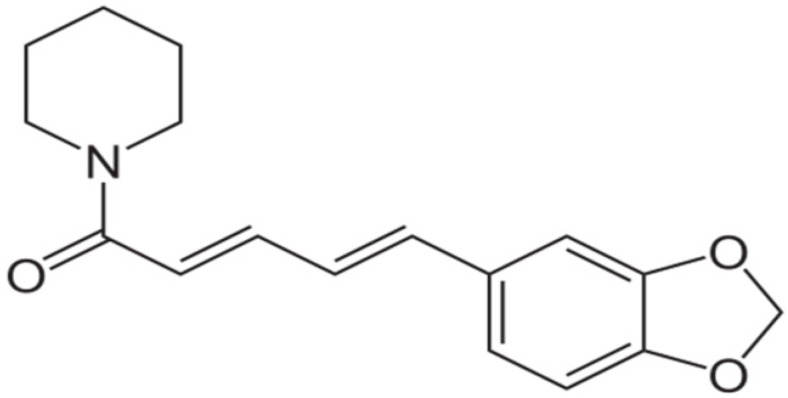	*Staphylococcus aureus* and *Bacillus subtilis* (MIC values of 225 µg/mL)	Alkaloids	Inhibition of efflux pump	[73,74]
**Berberine**	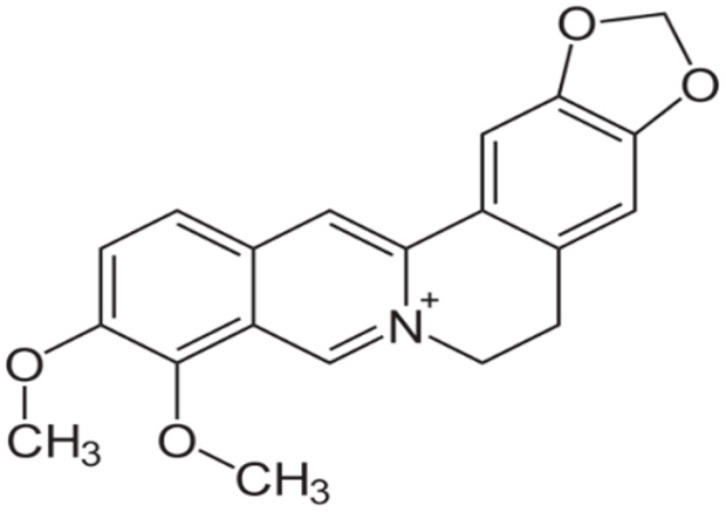	*Candida albicans* (MIC values ranging from 125 to 500 μg/ml)		DNA intercalation; inhibiting RNA polymerase, DNA gyrase, and topoisomerase IV, and IA; inhibiting protein biosynthesis, Inhibition of cell division	[75,76,77]
**Dictamnine**	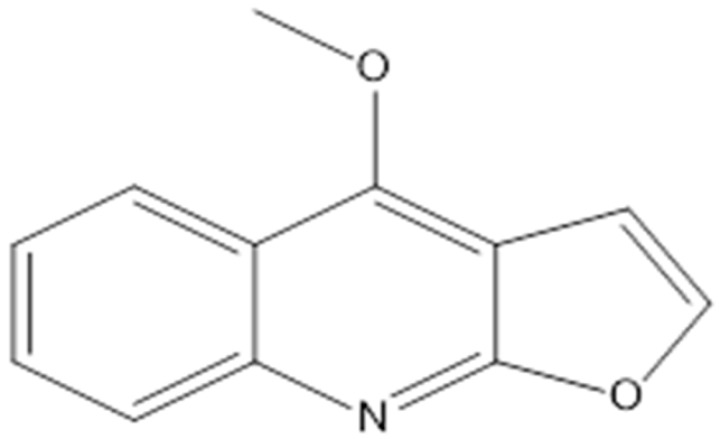	*Saccharomyces cerevisiae* (MIC value of 64 μg/ml)		Inhibiting type II topoisomerase	[78,79]
**Reserpine**	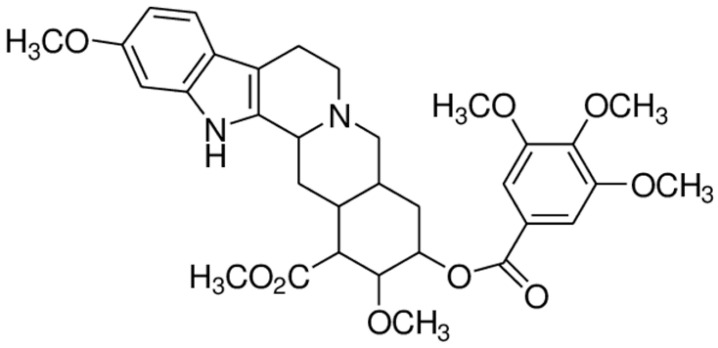			Inhibition of efflux pump	[80]
**Sanguinarine**	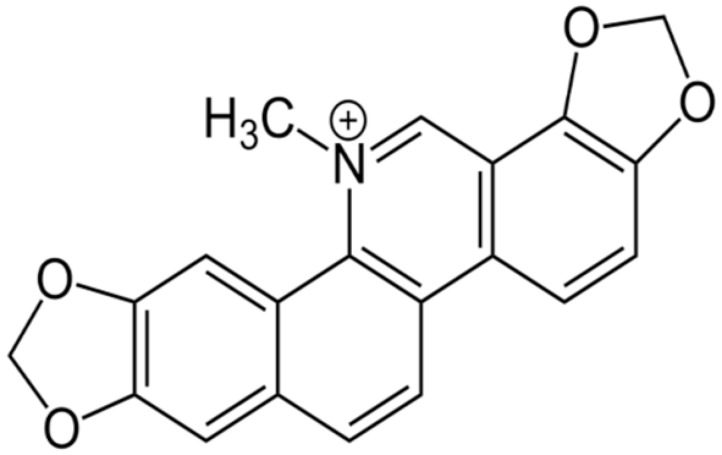	carbapenem-resistant *Serratia marcescens* (MIC90 of 32 μg/ml)		Inhibiting replication and transcription	[81,82]
**Chanoclavine**	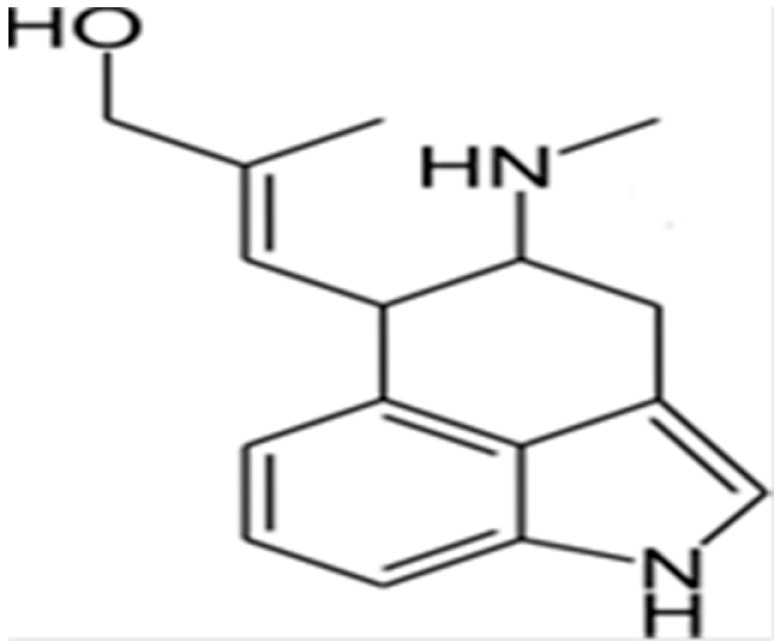			Inhibition of efflux pump	[83]
**Conessine**	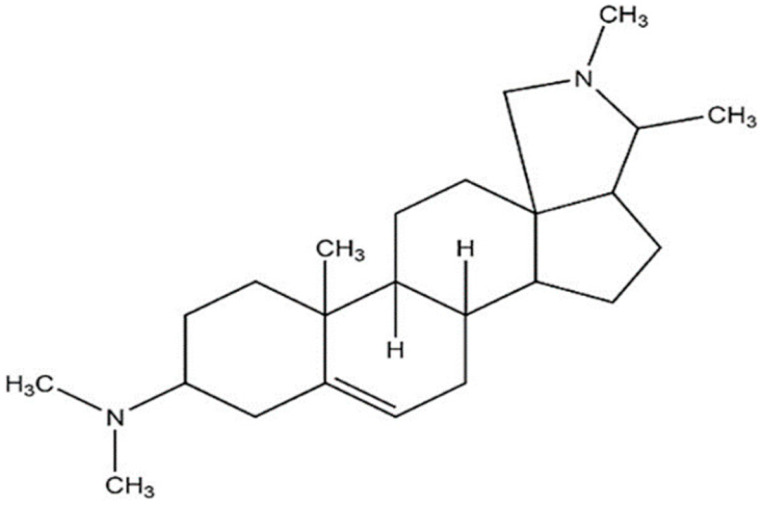	*Micrococcus luteus* ATCC 9341 (MIC value of 15.6 μg per disc)		Inhibition of efflux pump	[84,85]
**Chelerythrine**	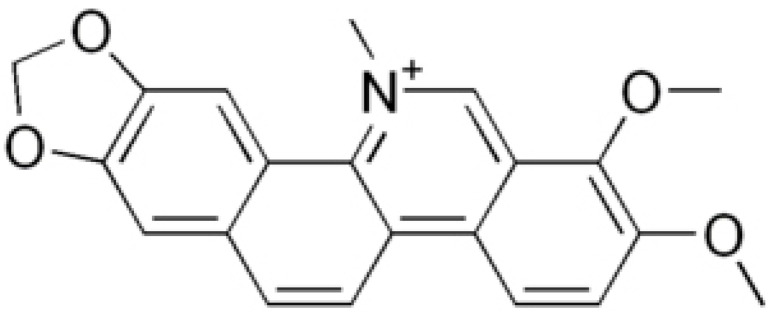	MRSA (MIC values ranged from 2 to 4 μg/mL) and extended-spectrum β-lactamases *Escherichia coli* (MIC values varied from 16 to 256 μg/mL)		Damaging the bacterial cells	[86,87]
**Matrine**	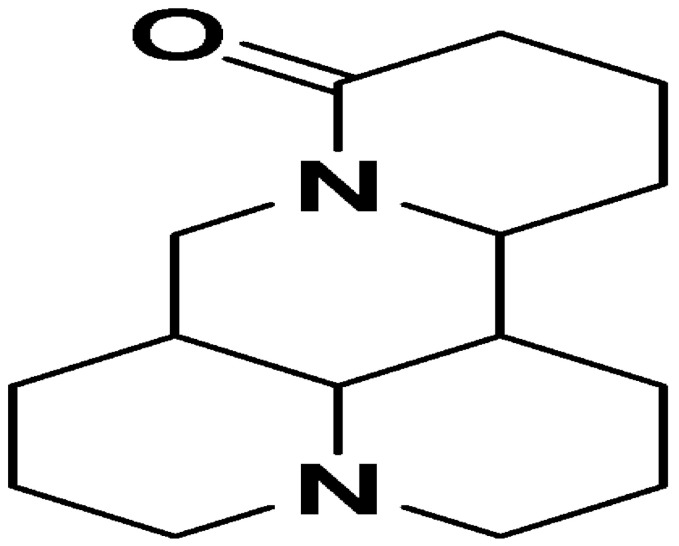	*E. coli* and *Bacillus subtilis* (12.5 μg/mL)		Inhibiting the synthesis of proteins	[88,89]
**Camptothecin**	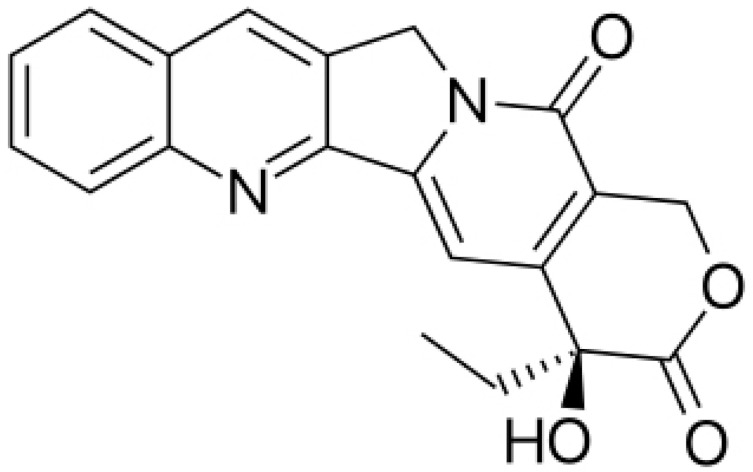			Cleaving the intermediate complex of DNA topoisomerase I	[90]
**Caffeine**	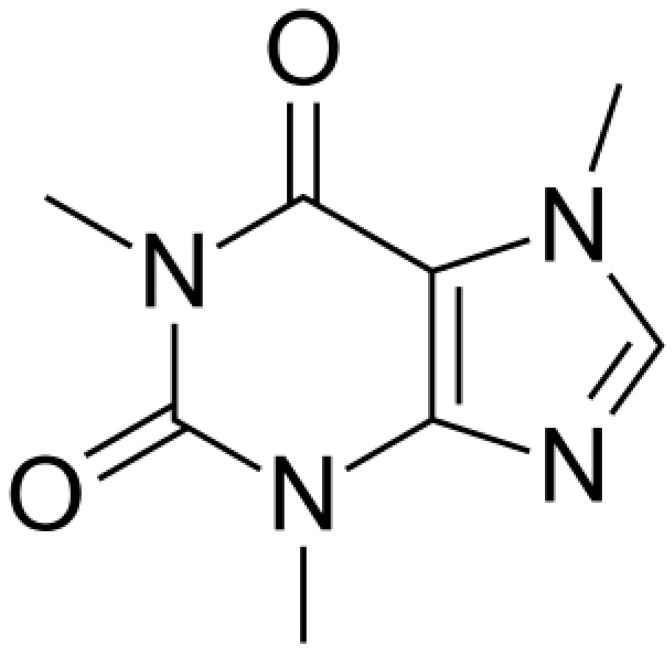	*P. aeruginosa* (MIC value of 200 μg/mL)		Interaction with the quorum sensing proteins and inhibiting biofilm formation	[91,92]
**Allicin**	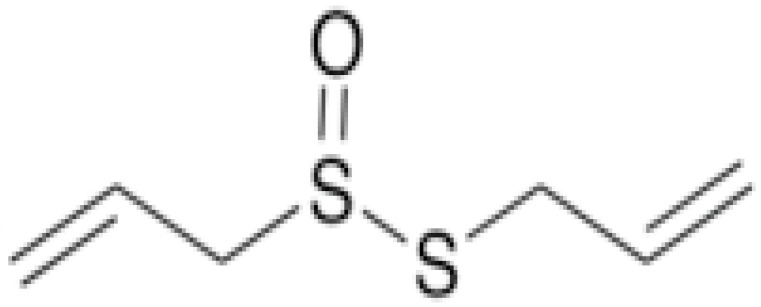	*C. albicans* (MIC value of 8 µg/ml)	Organosulfur	Inhibiting sulfhydryl-dependent enzymes, inhibiting the DNA and protein synthesis	[93,94]
**Ajoene**	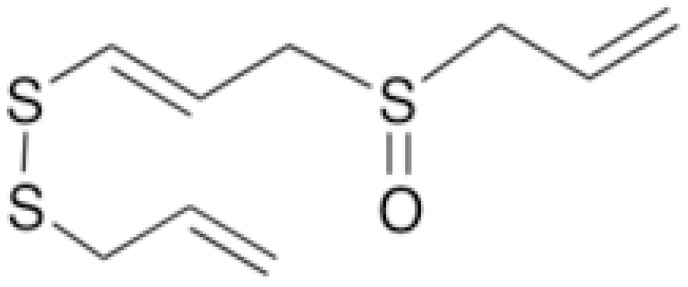	*Histoplasma capsulatum* (MIC values varied from 2.5 to 5 μg/mL)		Inhibiting sulfhydryl-dependent enzymes	[93,95]
**Isothiocyanates**	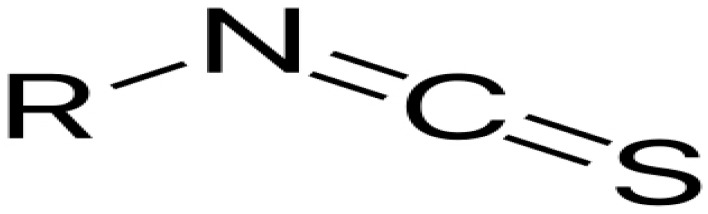			Attacking the sulfhydryl groups of enzymes, damaging the cell wall integrity, and leakage of cellular metabolites	[96]
**Diallyl Sulfides**	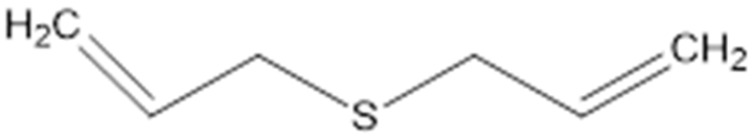	*C. albicans* (MIC value of 500 µg/ml)		Inhibiting glutathione (GSH) *S*-transferase (GST) activity. Interaction with the quorum sensing proteins and inhibiting biofilm formation	[97,98]
**Diallyl trisulfide (Allitridin)**	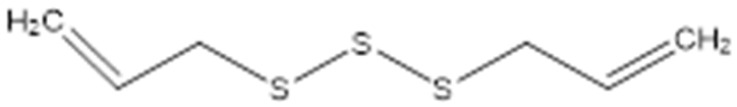			Destructing the bacterial cell membrane. Decreasing the activity of the bacterial membrane transporter system.	[99]
**Resveratrol**	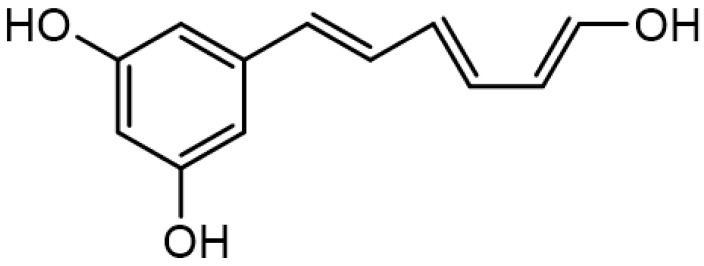	Multidrug resistant (MDR) Gram-negative (MICs ranging from 32 μg/mL to 128 μg/mL)	Polyphenolic compounds	Inhibition of efflux pump	[50,51]
**Baicalein**	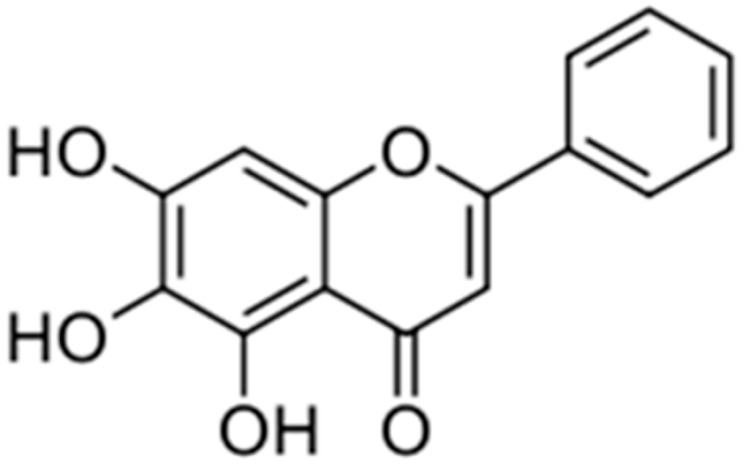	*S. typhimurium* (MIC value of 64 µg/ml)		Inhibition of efflux pump	[54,100]
**Biochanin A**	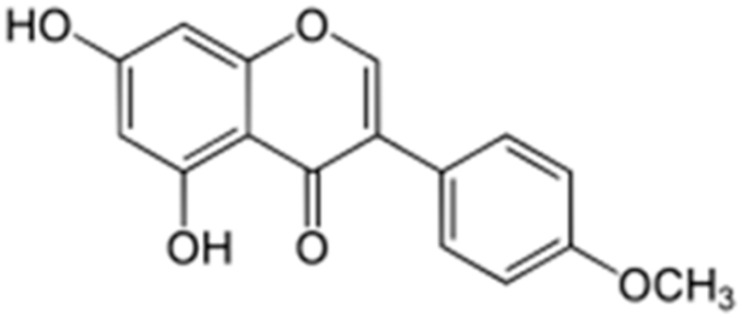	*S. aureus* (MIC values varied from 64 to 512 μg/mL)		Inhibition of efflux pump	[100,101]
**Chrysosplenol-D**	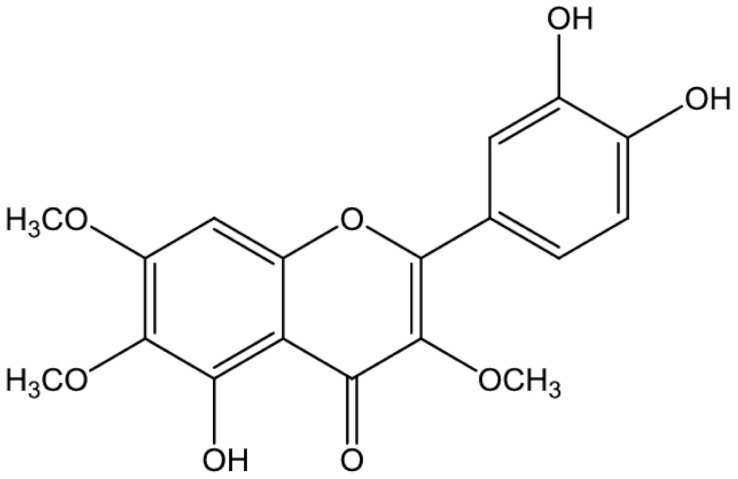			Inhibition of efflux pump	[102]
**Chrysoplenetin**	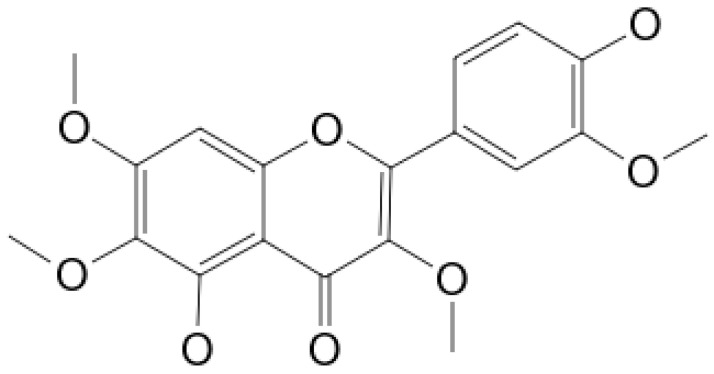			Inhibition of efflux pump	[102]
**Silybin**	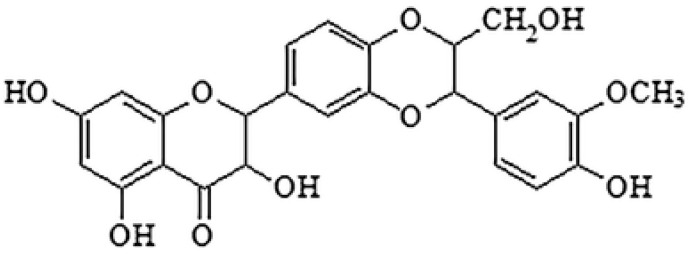			Inhibition of efflux pump	[103]
**Kaempferol**	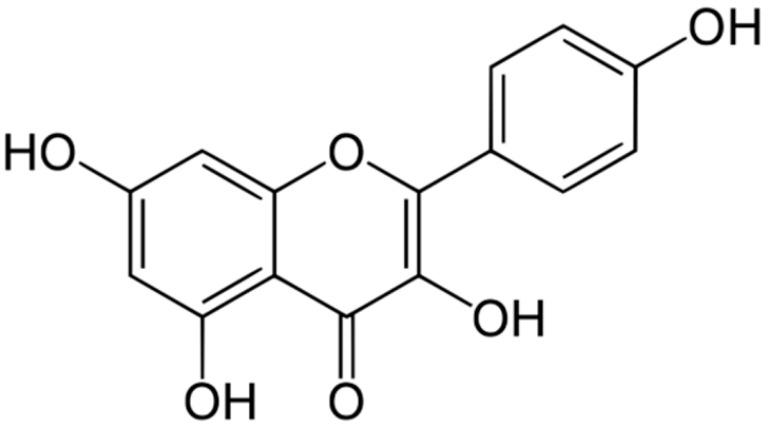			Inhibition of efflux pump	[104]
**Quercetin**	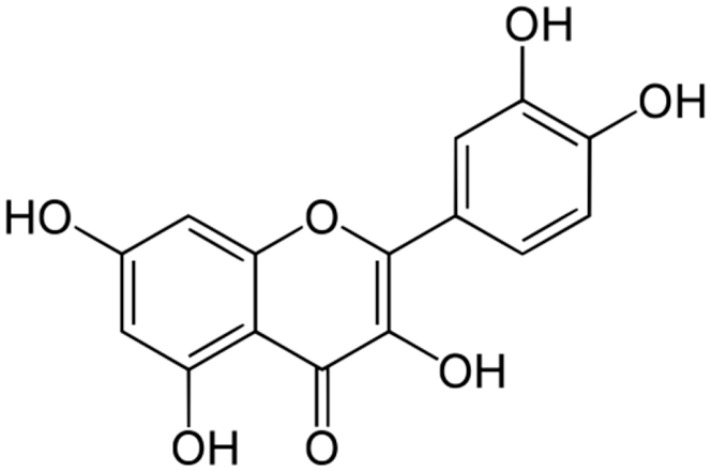	*Aspergillus fumigatus* (MIC values of 16–64 μM)		Inhibition of efflux pump, Interacting with some crucial enzymes such as β-lactamase, and cell membrane disruption	[45,105]
**Guttiferone-A**	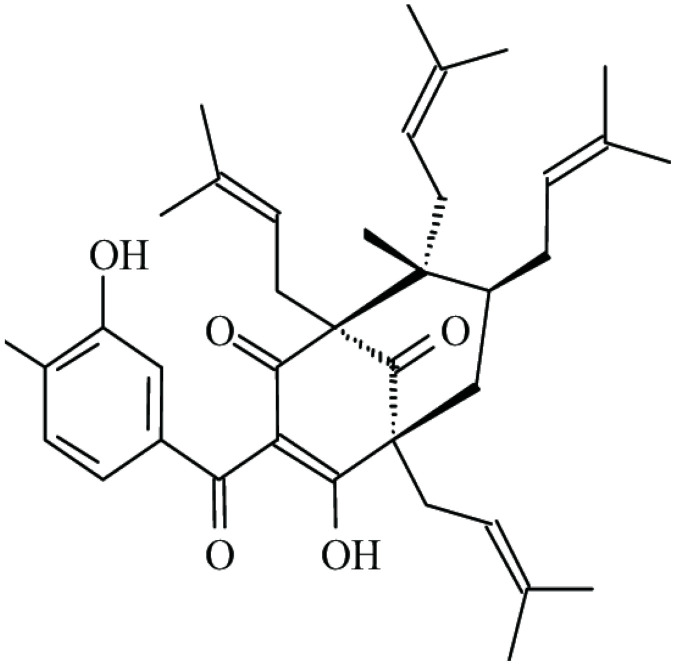			β-lactamase inhibition	[48]
**4-Butanylanisole**				β-lactamase inhibition	[49]
**Gallic acid**	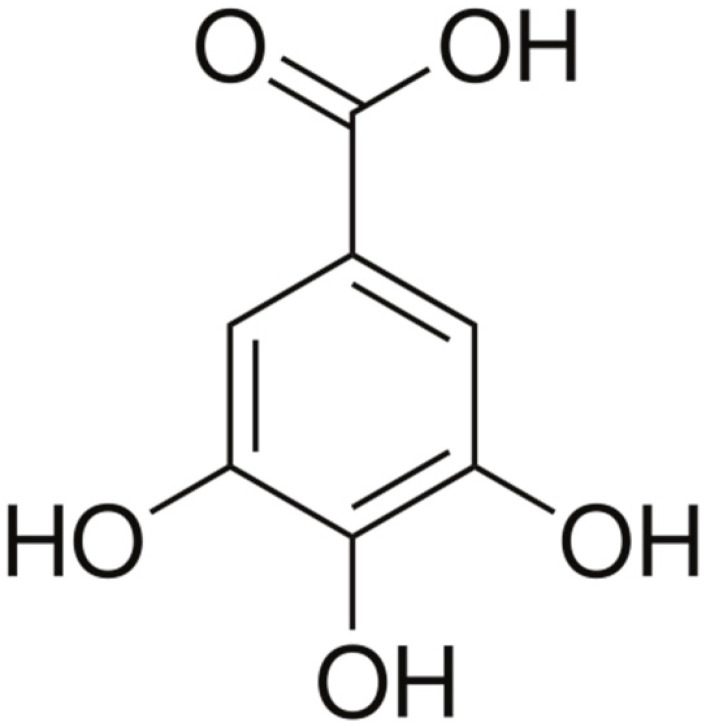			Cell membrane disruption, and Mg^2+^ Chelation	[106]
**Epigallocatechin gallate**	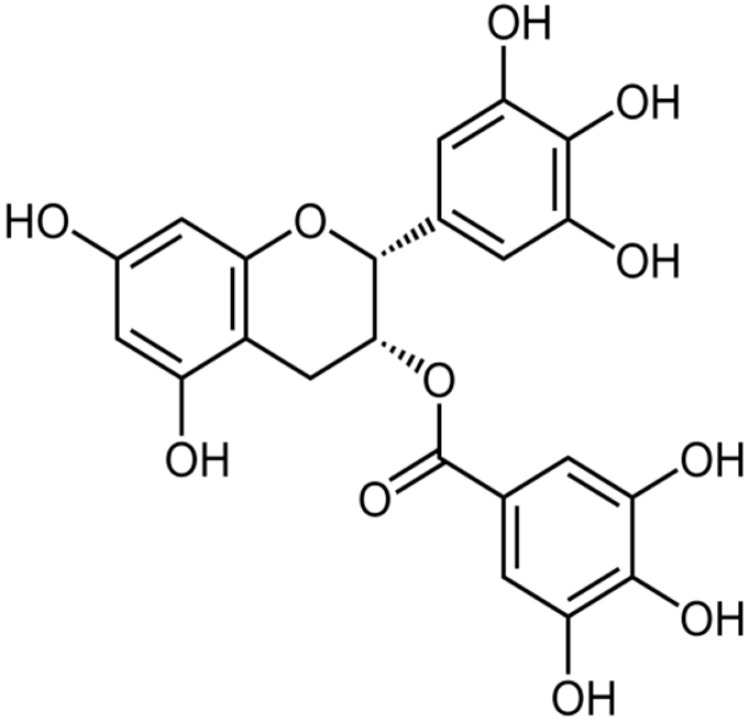	*S. aureus* (MIC values of 7.81–62.5 μg/mL)		Inhibiting the B subunit of DNA gyrase, penicillinase, and β-lactamase	[41,42,43,107,108]
**3-p-trans-Coumaroyl-2-hydroxyquinic acid**	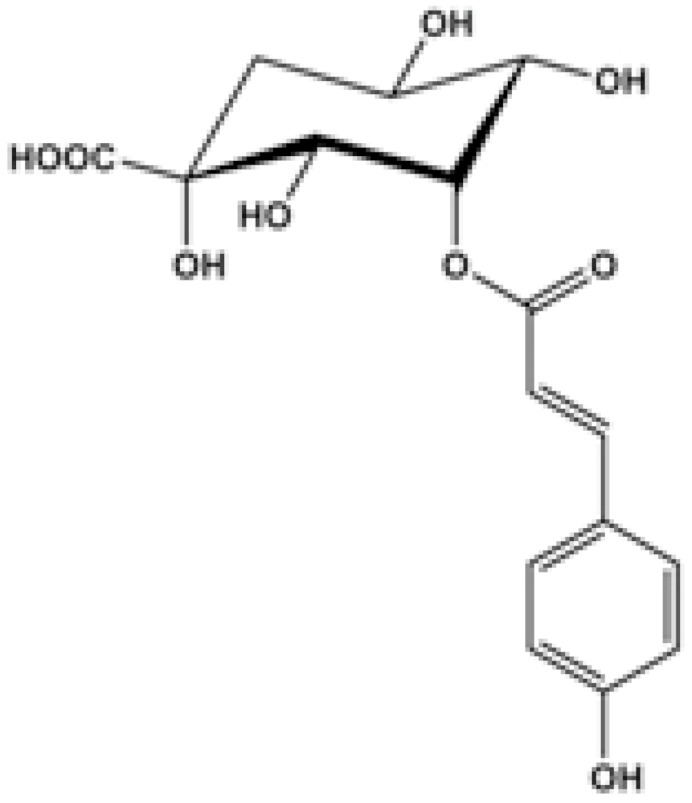			Damaging the cytoplasmic membrane	[109]
**Hydroxycinnamic acids** **(p-Coumaric, Caffeic, and Ferulic acids)**	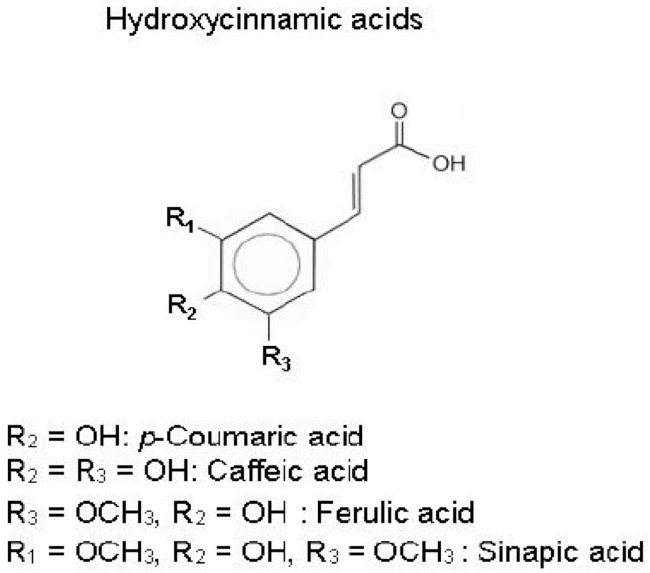			Interfering with membrane integrity	[110]
**Naringenin**	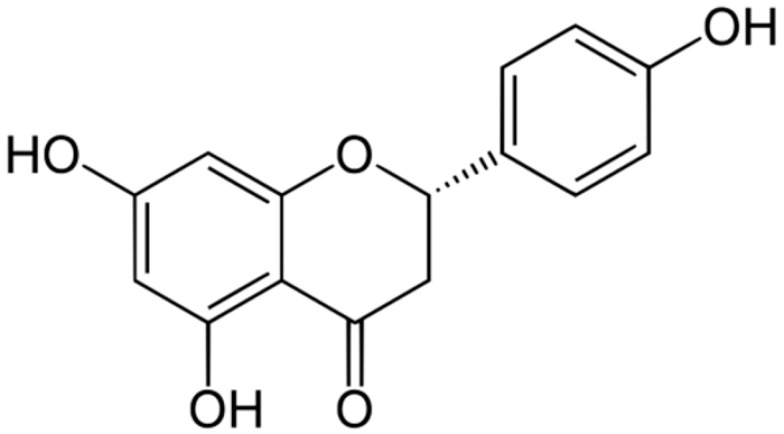			Interacting with some crucial enzymes	[111,112,113]
**Eriodictyol**	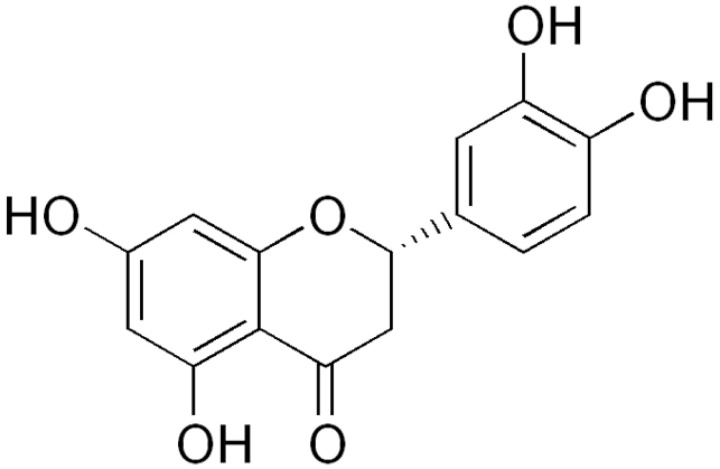	*Streptococcus mutans* and *P. aeruginosa* (MIC values of 1 mg/mL)		Interacting with some crucial enzymes	
**Taxifolin**	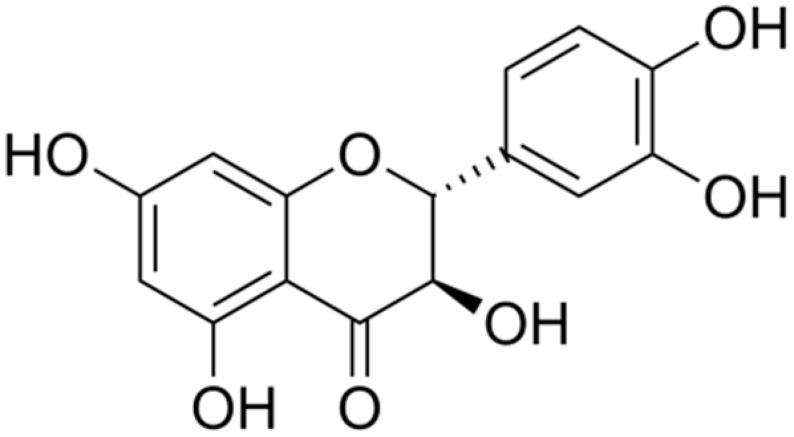	*Helicobacter pylori* (MIC = 625 μg/mL)		Interacting with some crucial enzymes	
**Curcumin**	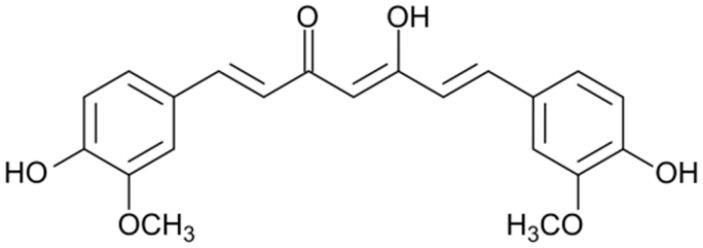	*Shigella dysenteriae* and *Campylobacter jejuni* (MIC values of 256 μg/mL)		Damaging the cell membranes	[114,115]
**Apigenin**	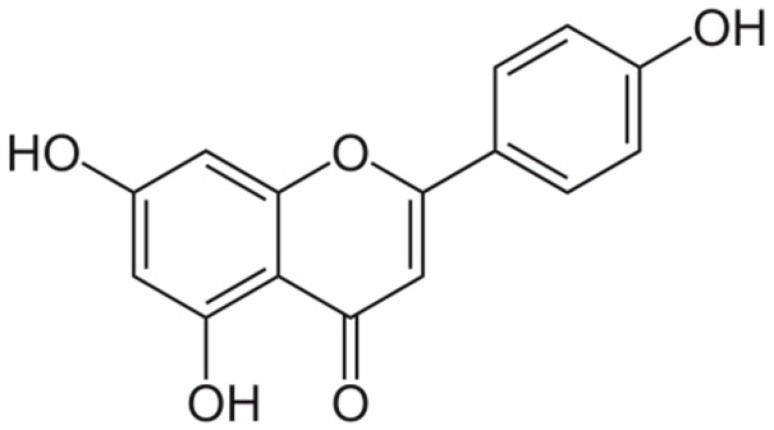			Interacting with some crucial enzymes	[116]
**Sophoraflavanone G**	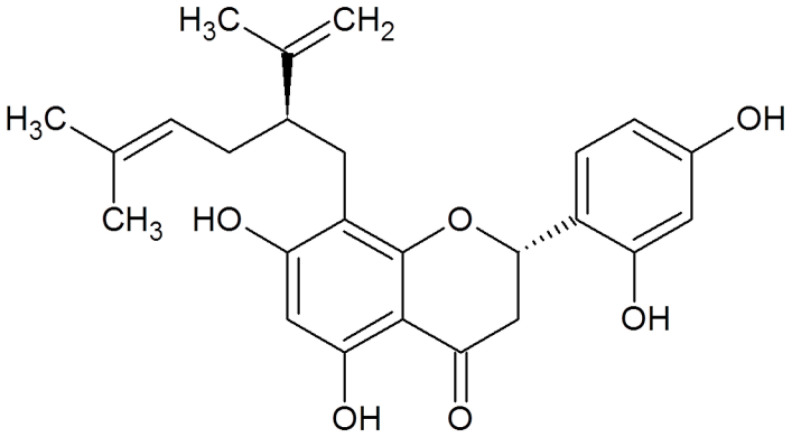	MRSA (MIC values of 0.5–8 μg/mL)		Interacting with peptidoglycan and inhibiting cell wall biosynthesis	[52,53]
**Acetosyringone**	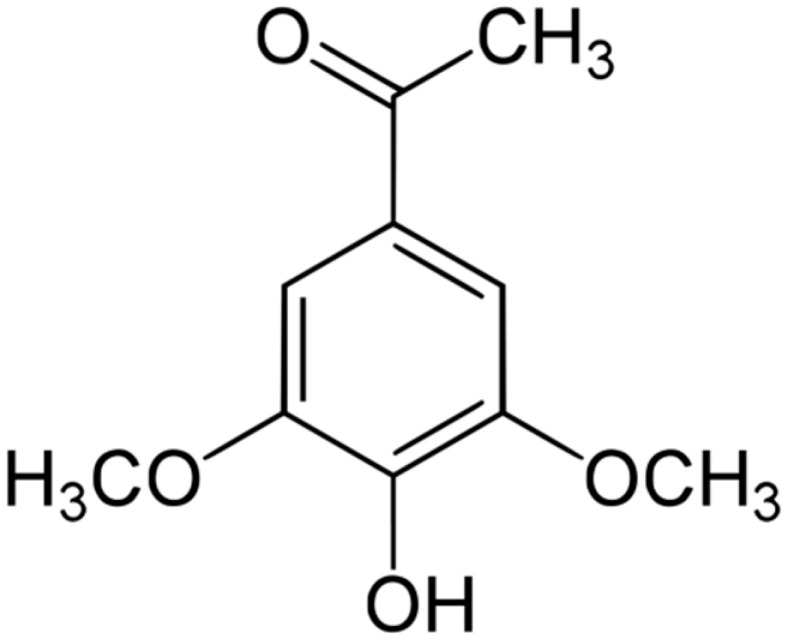	*S. cerevisiae* (MIC = 24 mM)		Depolarization of the bacterial cell membrane	[117,118]
**Chlorogenic acid**	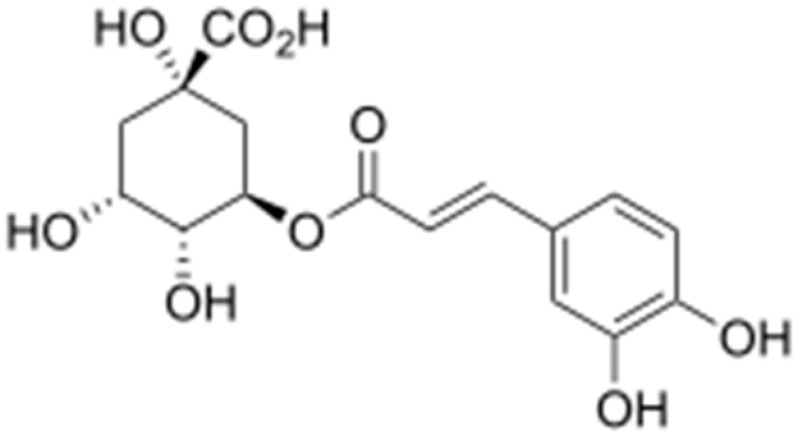	*Providencia alcalifaciens*, *Moraxella catarrhalis*, *S. aureus*, and *E. coli* ( MIC values of 60 to 100 μM)		Interacting with some crucial enzymes	[119]
**Galangin**	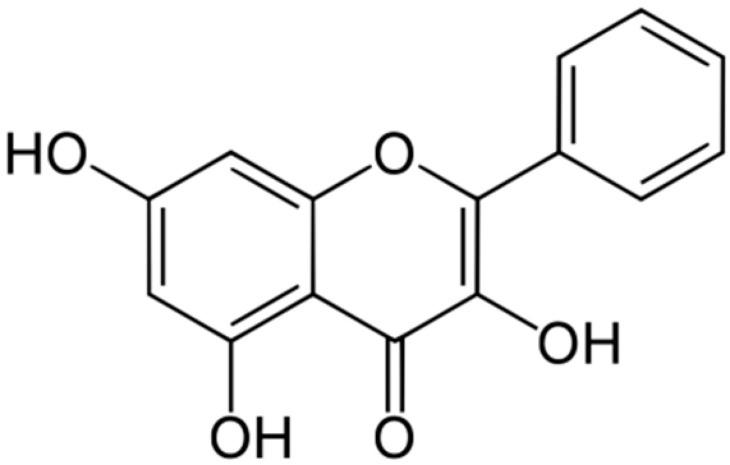	*S. aureus* (MIC = 32 μg/mL)		Damaging of the cytoplasmic membrane and inhibition of β-lactamase	[44,120]
**Genistein**	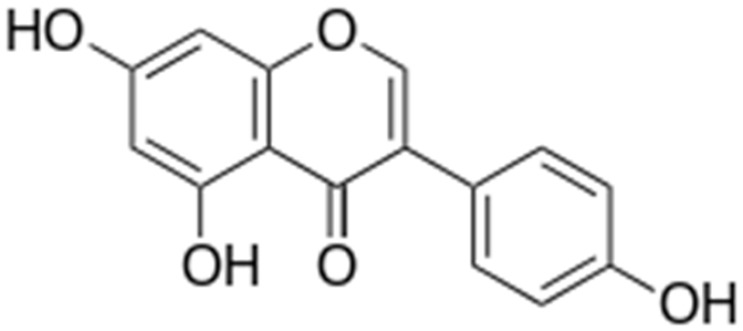			Inhibition of efflux pump	[121]
**Ononin**	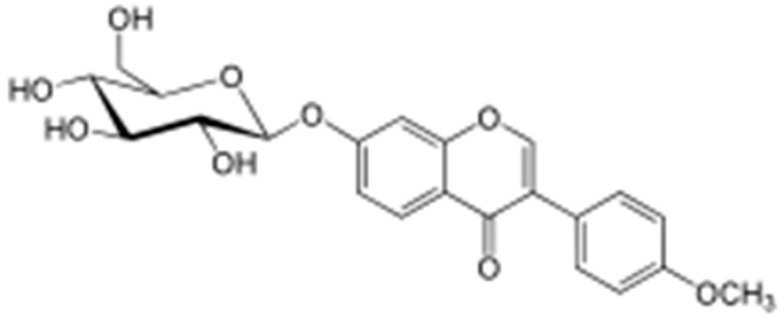				
**Tangeritin**	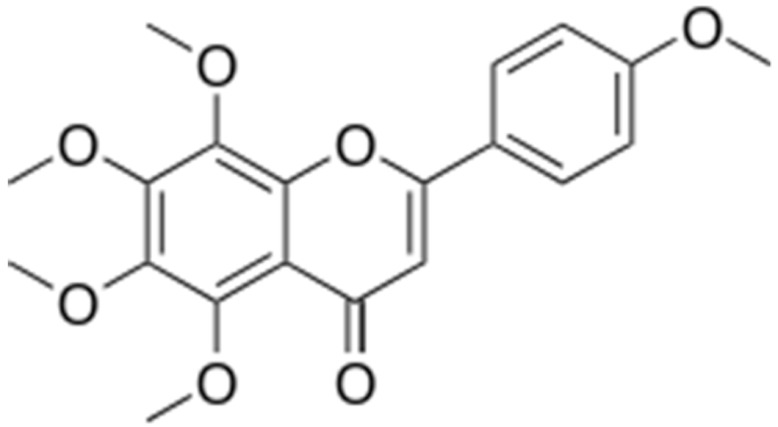			Cell membrane disruption, DNA gyrase inhibition, Reduced protein synthesis, Interacting with some crucial enzymes	[122]
**5,6,7,4’-** **Tetramethoxyflavone**	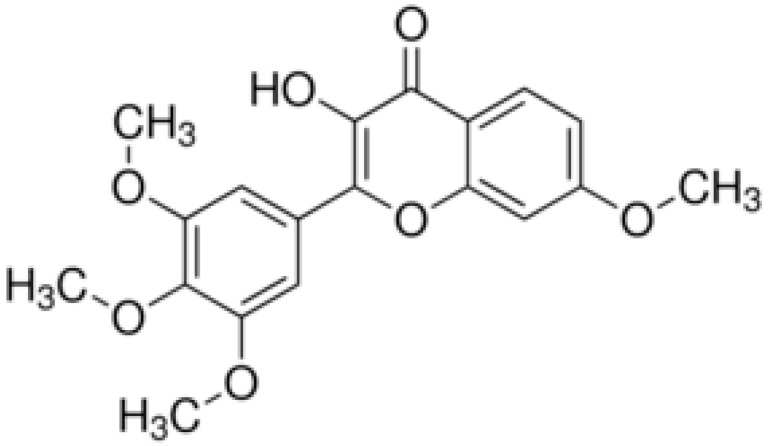			Cell membrane disruption, DNA gyrase inhibition	
**Chrysin**	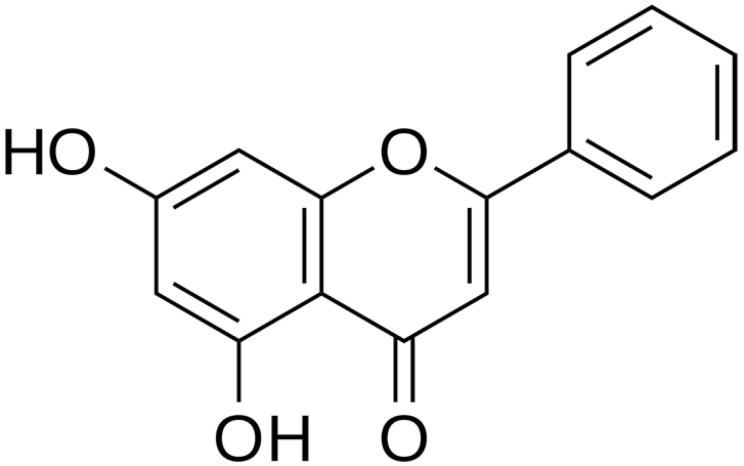	*H. pylori* (MIC = 6.25 μg/mL)		Cell membrane disruption, DNA gyrase inhibition	[123,124]
**Luteolin**	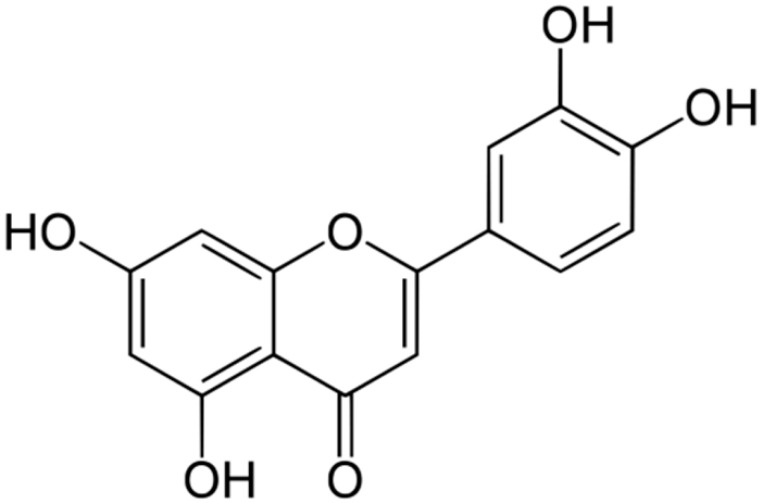	*S. aureus* (MIC = 16–32 μg/mL) and *Listeria monocytogenes* (MIC = 32–64 μg/mL)		Cell membrane disruption, DNA gyrase inhibition, Type III secretion inactivation, Interacting with some crucial enzymes	[125,126,127]
**Myricetin**	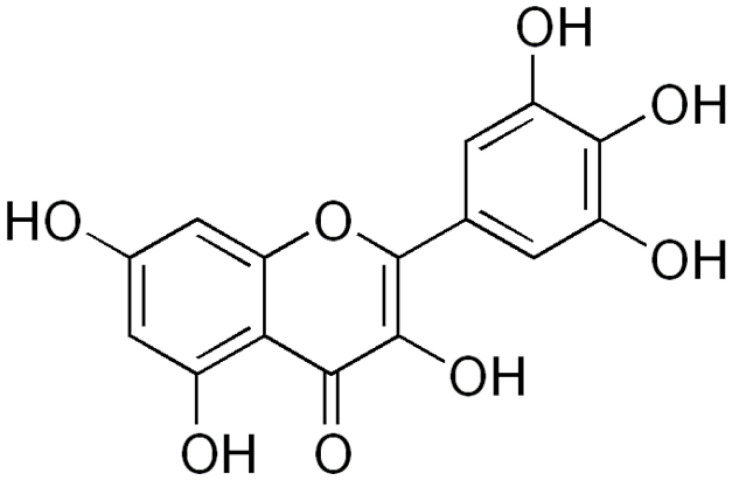	*S. aureus* (MIC = 256 μg/mL)		DNA gyrase inhibition, Type III secretion inactivation, Interacting with some crucial enzymes	
**Nobiletin**	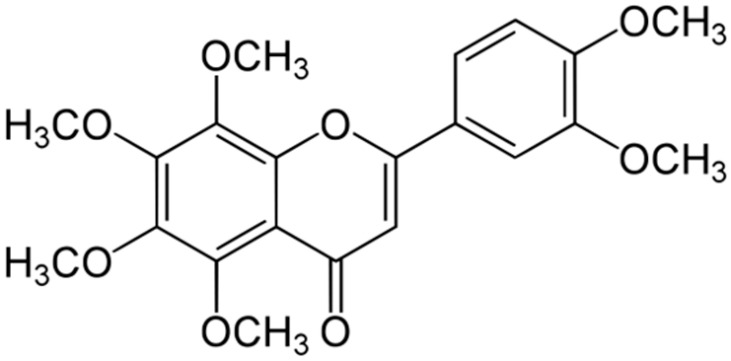			Cell membrane disruption, DNA gyrase inhibition, Reduced protein synthesis, Interacting with some crucial enzymes	
**Totaral**	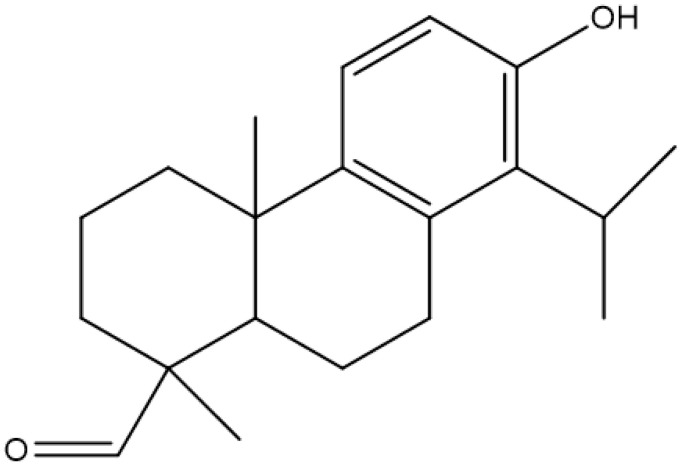			Reduced expression of enterotoxins, multi-drug efflux pump inhibitor	[128]
**Tannic acid**	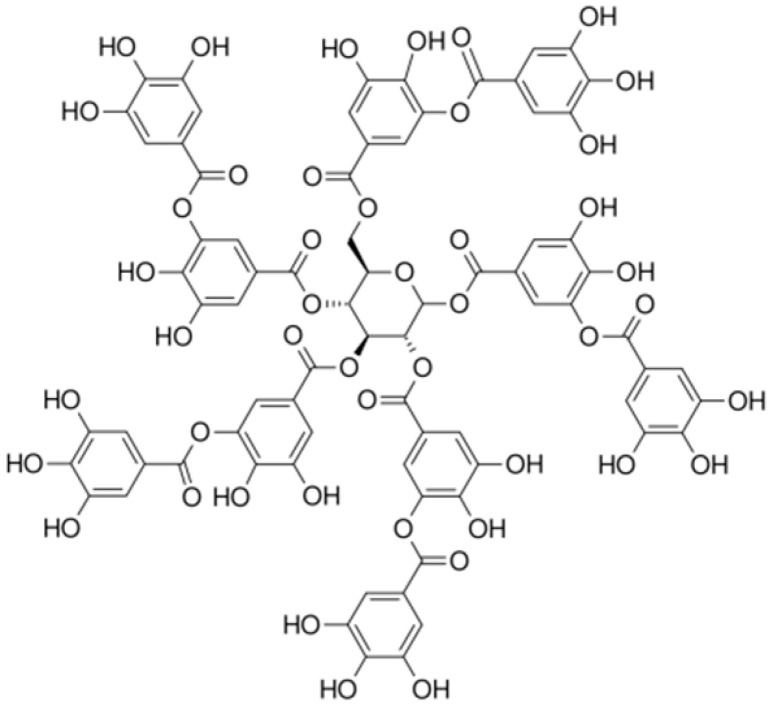	*S. aureus* (MIC = 512 μg/mL)		Ion binding	[129,130]
**(+)-Catechin**	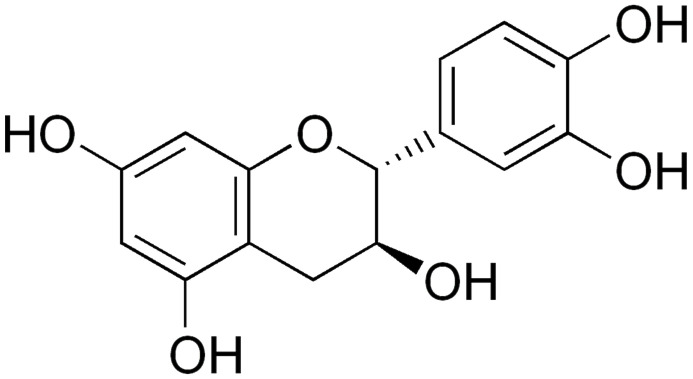	MRSA (MIC = 78.1–156.2 μg/ml)		Inhibition of bacterial gene expression	[131,132]
**Aegelinol**	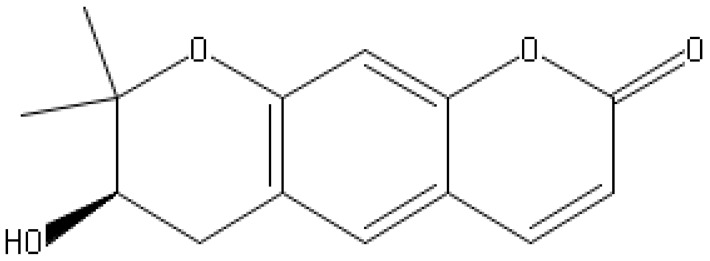	*S. aureus*, *S. thypii*, *Enterobacter cloacae* and *E. earogenes* (MIC = 16 μg/mL)	Coumarins	Cell membrane Disruption	[68,133]
**Agasyllin**	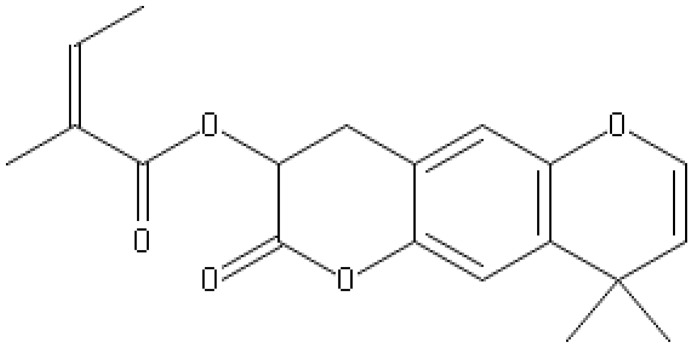	*S. aureus*, *S. thypii*, *Enterobacter cloacae* and *E. earogenes* (MIC = 32 μg/mL)		Cell membrane Disruption	
**Osthole**	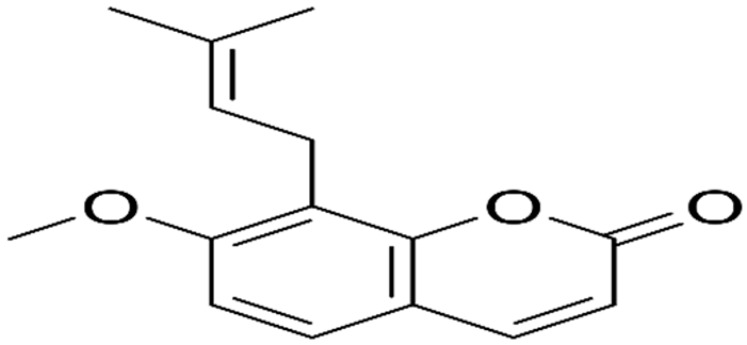			DNA gyrase inhibitor	[134]
**Clorobiocin**	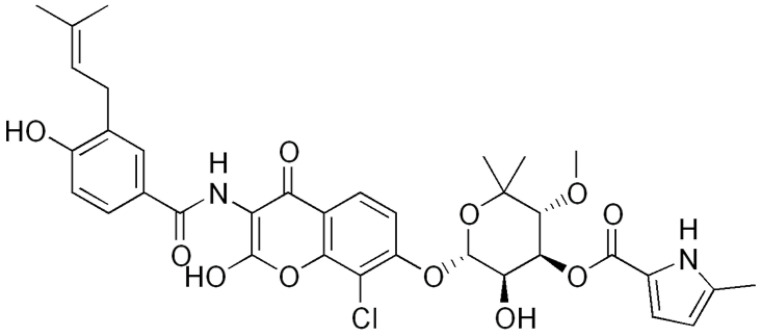			Inhibiting of DNA topoisomerase type II (DNA gyrase)	[135,136,137]
**Novobiocin**	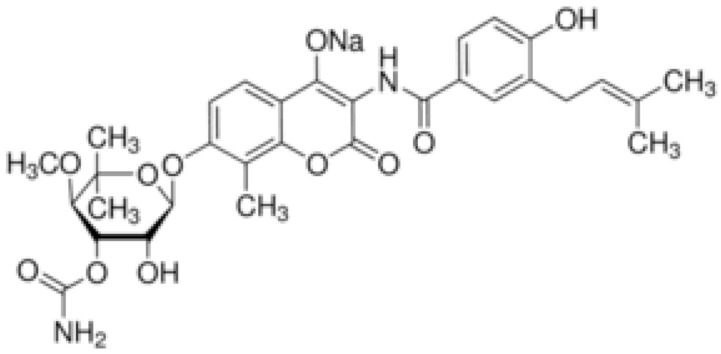	*S. aureus* and *S. gallinarum* (MIC = 2 and 0.25 mg/L)			
**Coumermycin A1**	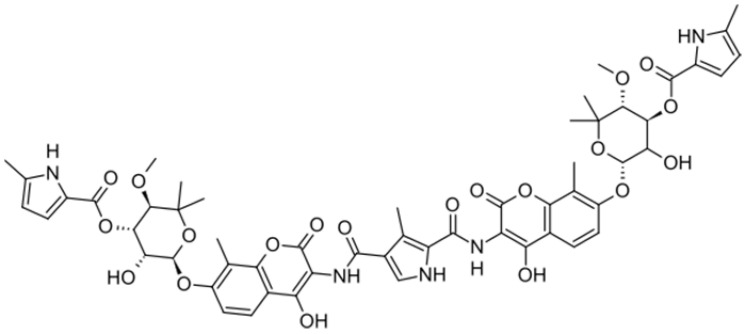				
**Bergamottin**	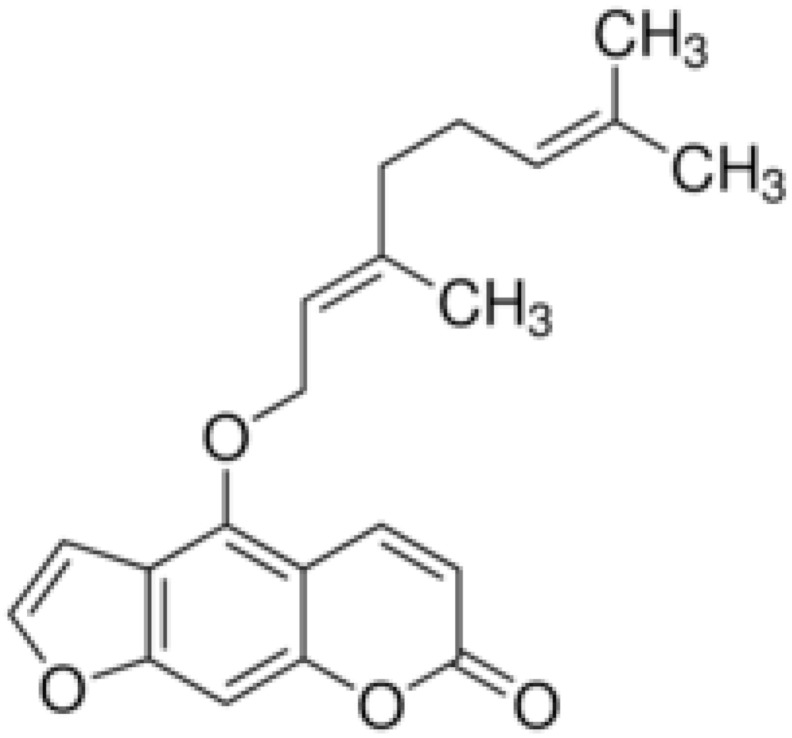			Inhibition of efflux pump	[138,139]
**6-Geranyl coumarin**	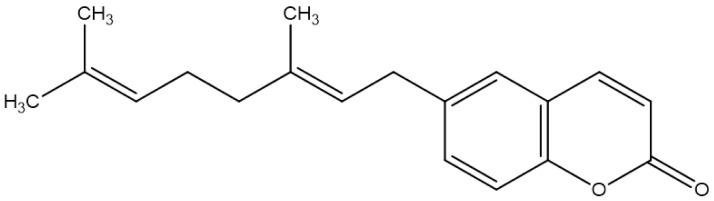				
**Gallbanic acid**	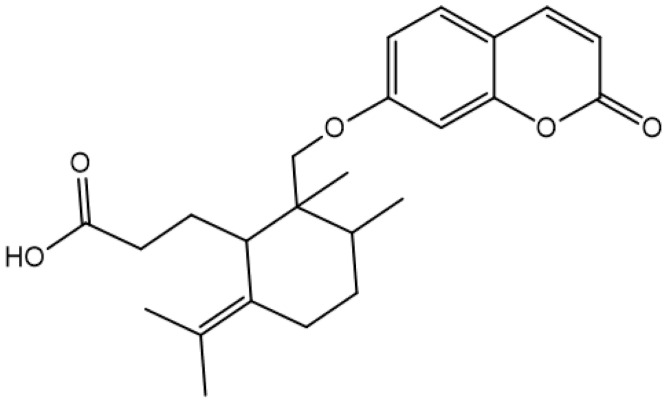				
**Daphnetin**	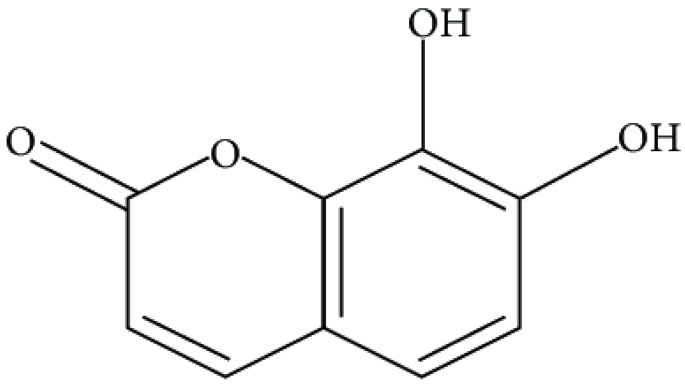	*P. fluorescens* and *Shewanella putrefaciens* (MIC values were 0.16 and 0.08 mg/mL, respectively)		Cell membrane Disruption, Type III secretion inactivation	[140,141,142]
**Esculetin**	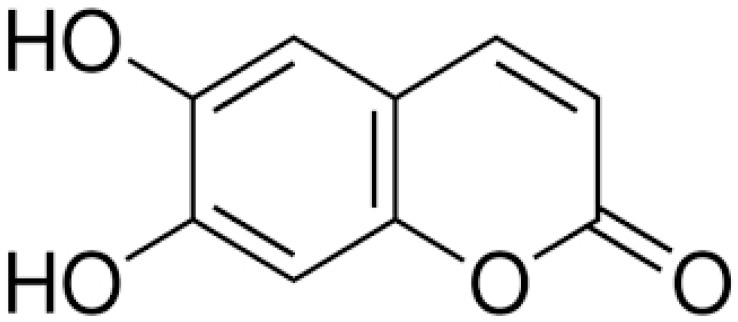	*Ralstonia pseudosolanacearum* (MIC = 125 mg/mL)			
**Umbelliferone**	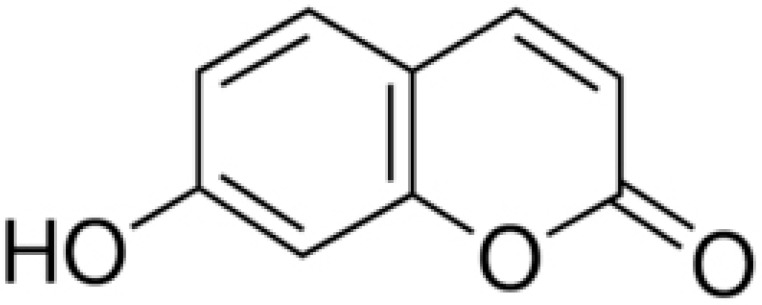	*R. pseudosolanacearum* (MIC = 325 mg/mL)			
**Carvacrol**	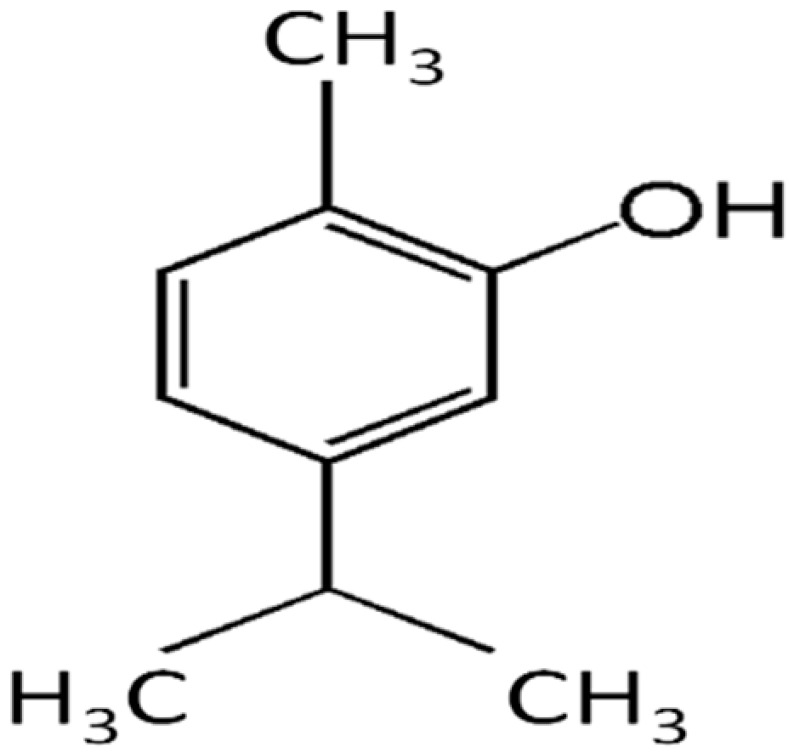	*Streptococcus pyogenes* (MIC = 125 μg/mL)	Terpenes	Disrupting cell membrane integrity, Inhibition of efflux pump	[143,144,145,146,147]
**Thymol**	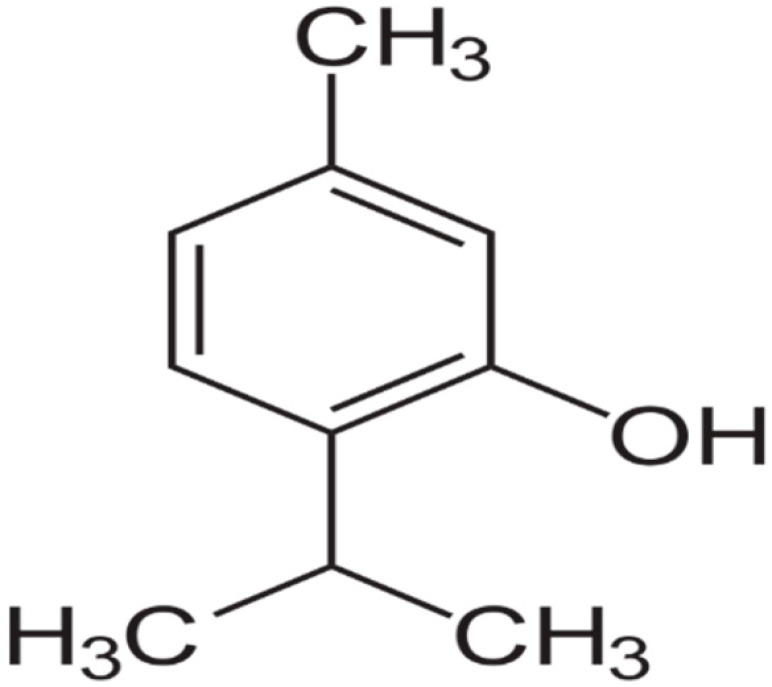	*B. cereus* (MIC = 0.625 mg/mL)			
**Soyasaponin V**	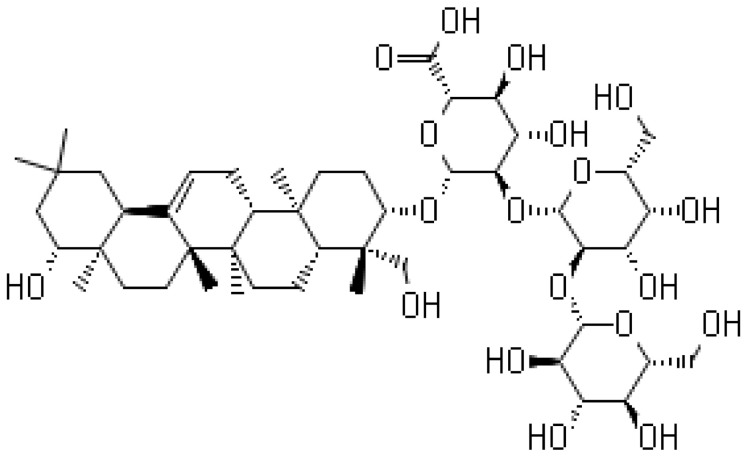			Inhibition of the New Dehli Metallo-β-lactamase 1	
**Eugenol**	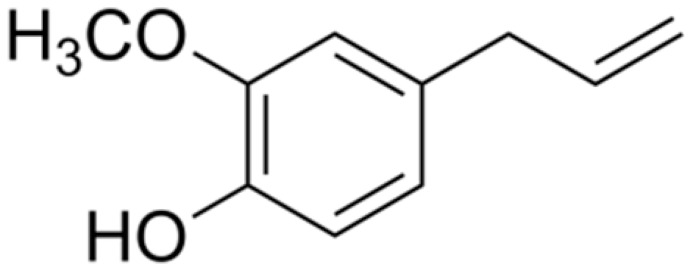	*E. coli* (MIC ranging from 0.0312 to 8 μg/mL)		Disrupting cell membrane integrity	
**α-Pinene**	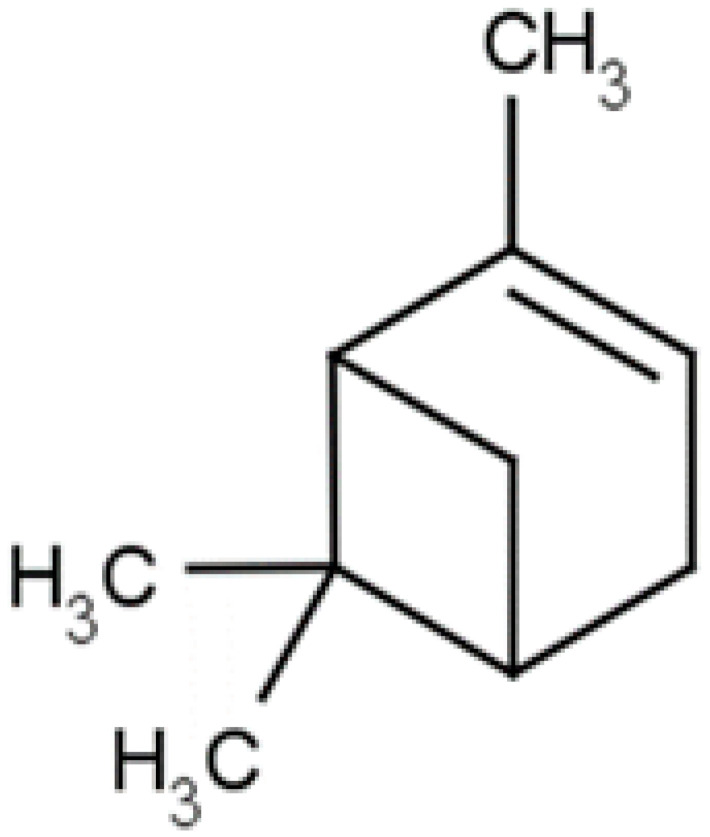	*H. pylori* ( MIC ranged from 275 to 1100 μg/mL)			
**Limonene**	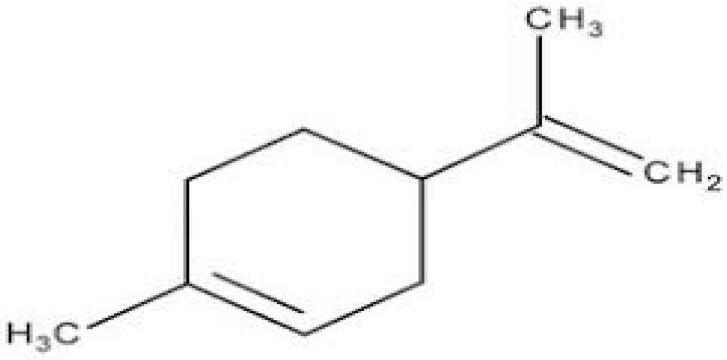	Standard *S. aureus* (MIC = 256 μg/mL) and resistant *P. aeruginosa* (MIC = 512 μg/mL)			
**Menthol**	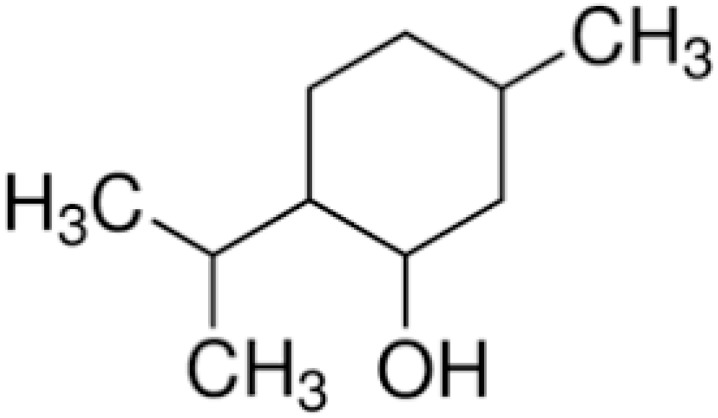	*C. albicans* (MIC 90 were 1.6 to 25 μg/mL)			
**Farnesol**	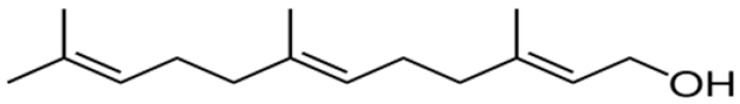	Lactobacillus spp. (MIC = 1500 µM)		Disrupting cell membrane integrity	[148,149,150,151,152,153]
**Nerolidol**	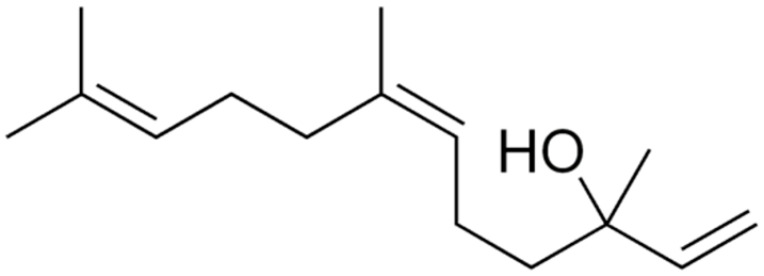	*S. aureus* (MIC = 1 mg/mL), *S. mutans* (MIC = 4 mg/mL), *P. aeruginosa* (MIC = 0.5 mg/mL), and *K. pneumoniae* (MIC = 0.5 mg/mL).			
**Carvone**	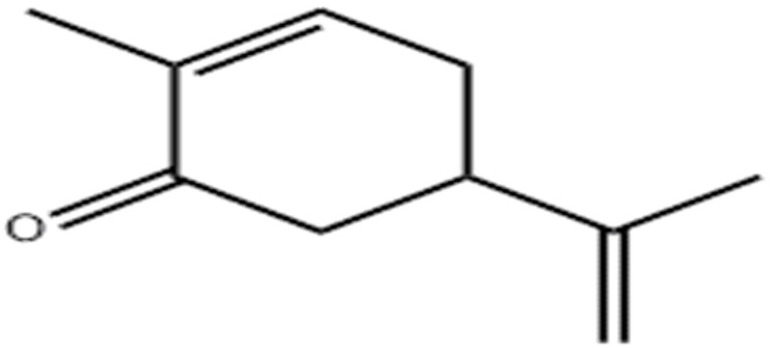			Inhibiting the transformation of cellular yeast to the filamentous	[154]
**Ursolic acid**	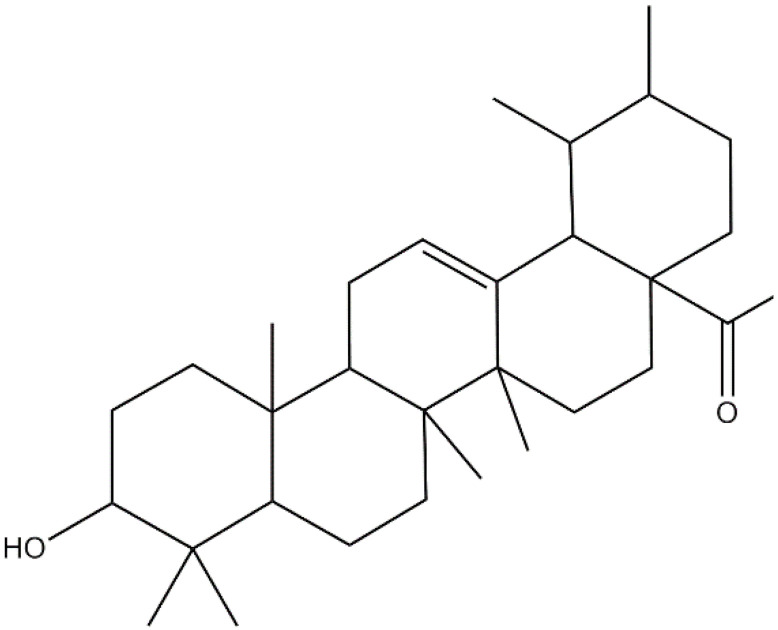	Carbapenem-resistant *E. cloacae* (MIC = 0.1 mg/mL)		Disrupting cell membrane integrity and inhibition of β-lactamase	[155,156]
**α-Amyrin**	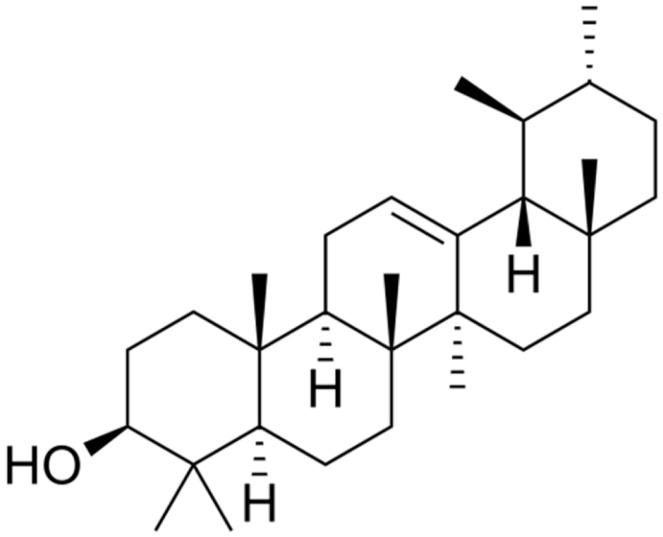				[157]
**Cinnamaldehyde**	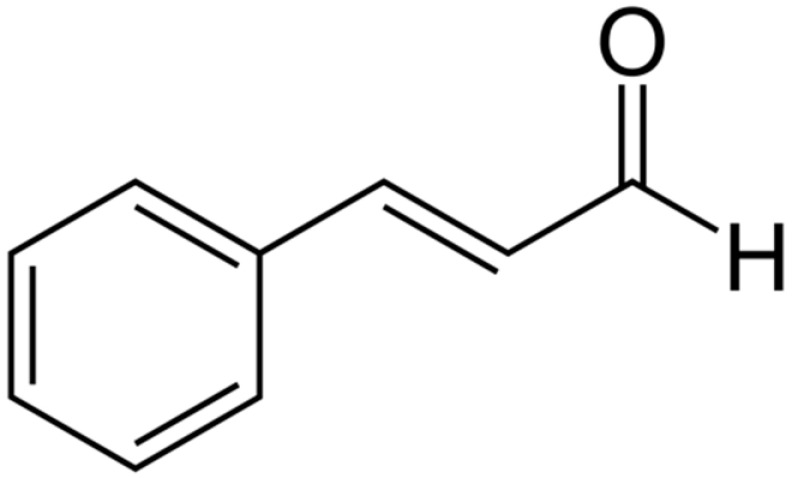	*E. coli* (MIC = 780 µg/mL)		Disrupting cell membrane integrity, Decreasing membrane potential, and metabolic activity	[158,159]
**Artemisinin**	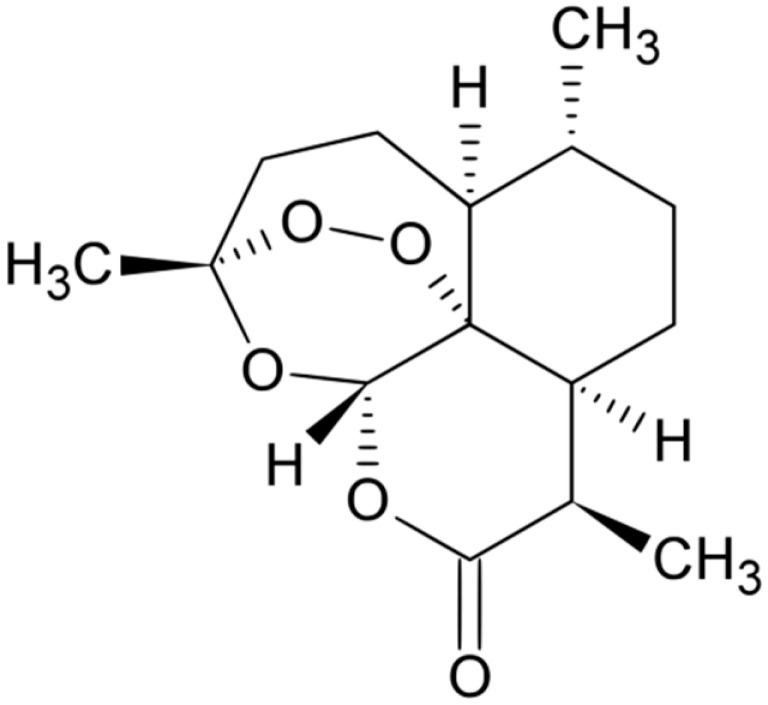			Free radicals formation	[160]
**Linalool**	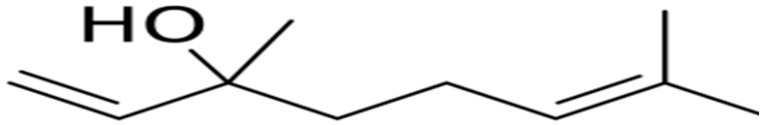	*P. aeruginosa* (MIC = 431 μg/mL)		Disrupting cell membrane integrity, changing in the nucleoid morphology, and interfering with cellular respiration	[161,162,163]
**Sabinene**	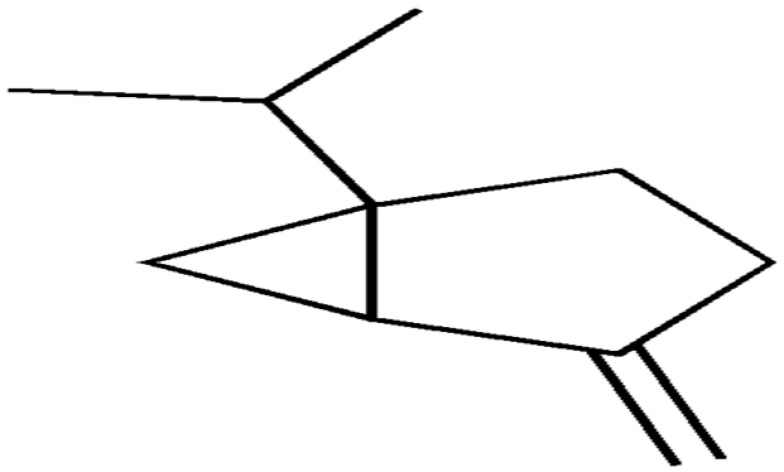	Multi drug-resistant strains (MIC ≥ 1024 μg/mL)		Disrupting cell membrane integrity and inhibiting DNA synthesis	[164,165]
**α-Terpineol**	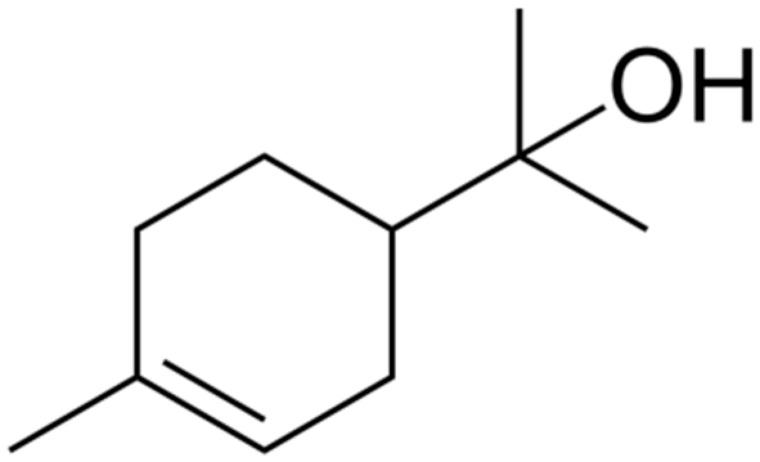	*E. coli* (MIC ≥ 0.78 μg/mL)		Lossing membrane-bound autolytic enzymes, the cytoplasm leakage and inability to osmoregulate	[166,167]
**Citronellol**	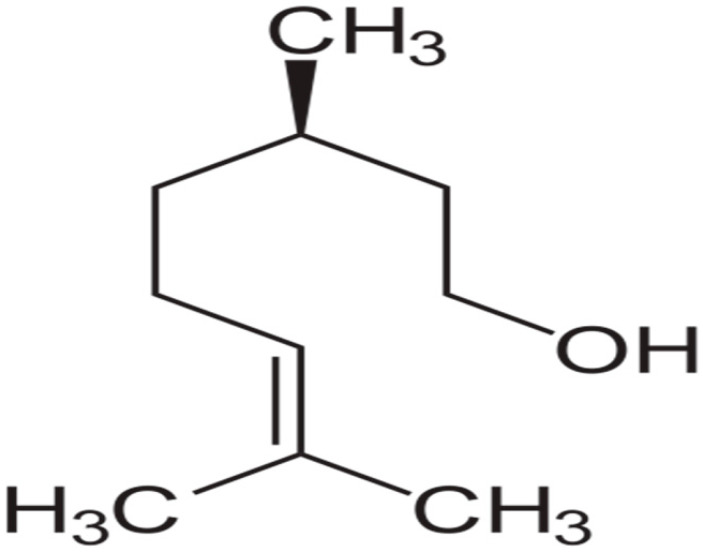	*Trichophyton rubrum* (MIC values from 8 to 1024μg/mL)		Deteriorating membrane integrity	[168,169]
**α-Bisabolol**	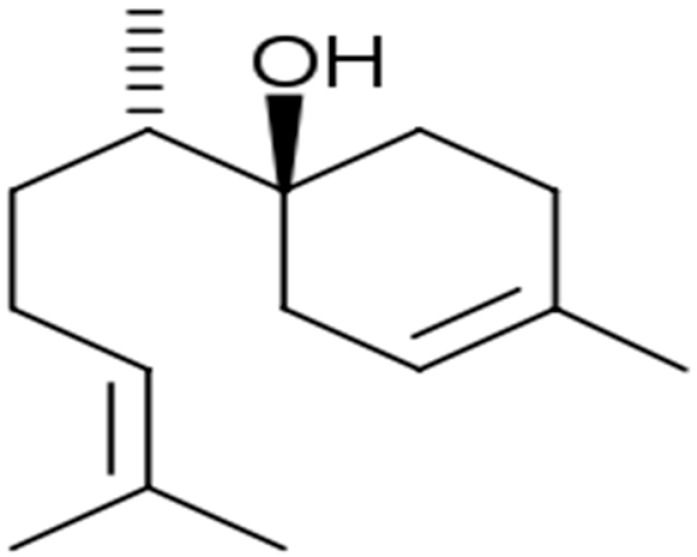	*Propionibacterium acnes* and *S. epidermidis* (MIC = 75 and 37.5 μg/mL)		Disrupting cell membrane integrity	[170,171]

Gram-positive bacteria are commonly more susceptible to terpenes than Gram-negative ones [172].

Catteau et al. [157] obtained a dichloromethane extract from the leaf of the shea butter tree (*Vitellaria paradoxa*) in which the triterpenic acids, ursolic acid (UA), and oleanolic acids (OA) were identified as major constituents. Both of these compounds, in the presence of β-lactams, restored the efficacy of the antibiotics against MRSA. UA proved more effective than OA with MIC values of 8–16 mg/L and 32–128 mg/L for UA and OA, respectively. Their ability to inhibit β-lactamase of living bacteria was observed among the different mechanisms, but this was not evident in bacteria lysates, suggesting an indirect mechanism was involved in the inhibition.

Horie et al. [144] reported the synergistic effects of soybean saponins on the antimicrobial activity of β-lactam antibiotics against *S. aureus* strains producing β-lactamases. In the presence of crude saponins, there was a significant decrease in the activities of β-lactamases, including the New Dehli Metallo-β-lactamase 1 (NDM-1). The latter had not been reported to be inhibited by any of the current β-lactamase inhibitors. The individual saponin components examined showed that the presence of 200 µg/mL soysaponin V significantly (*p* < 0.001) inhibited NDM-1 compared to equivalent levels of either soysaponin I or soysaponin B. The combination of soy saponins and β-lactam antibiotics was proposed as a new therapeutic modality, particularly against bacteria encoding NDM-1. Table 1 shows the most prevalent natural antibacterial compounds with the related mechanism of action.

## 4. Preclinical and Clinical Studies on Antibacterial Effects of Phytochemicals

Many antimicrobial herbal medicines show antimicrobial activities that may serve as possible treatment alternatives to conventional antimicrobial regimens for infections sensitive to conventional antibacterial agents and resistant strains of microorganisms. As part of the efforts to broaden the employment of herbal medicines to treat infectious diseases, preclinical and clinical testing guidelines for phytochemicals should ensure consistency in formulation, efficacy, and safety. Finally, phytodrugs, original medications obtained from medicinal plants, have been preclinically tested and then licensed in a particular country through clinical trials. They are usually a complex of two or more biologically active constituents.

Considering the vast number of natural compounds that have been identified in the last two centuries, only a very small number of them have already been examined under clinical trials. Also, at the same time, hundreds of similar projects are being performed in preclinical evaluations in the clinical laboratory. Table 2 summarizes information about some of the important herbal-derived products as antibacterial agents in human health care.

### 4.1. Concentrated Herbal Extract Granules TRA

Urinary tract infections (UTIs) are among the most common infections and are a frequent reason for hospitalization and antimicrobial therapy. Increasing antimicrobial resistance has stimulated interest in the non-antibiotic prevention of UTIs [173]. The standardized concentrated herbal extract granules TRA were used in the “Tokoro Combination” and “Rehmannia and Akebia Formula” The product was prepared in small granules, including concentrated herbal extract granules of "Tokoro Combination"(50%) and “Rehmannia and Akebia Formula" (50%). The Ministry of Health and Welfare in Taiwan has already approved both medicines as ethical drugs. The major components in this product were diosgenin, yamogenin, betulin, oleanolic acid, hederagenin, akeboside, β-sitosterol, stigmasterol, inositol, catalpol, glycyrrhizin, etc. Clinical trials.gov identifier (NCT number) of this study is “NCT04272437”.

### 4.2. Uva Ursi Extract

*Arctostaphylos uva-ursi* (bearberry extract arbutin) has been traditionally used to treat UTI symptoms. Antiseptic and antimicrobial properties of uva-ursi have been proved, attributed to hydroquinones and tannins. *Uva-ursi* is concentrated in the urine and has shown to be effective against bacteria causing UTIs 173]. It is safe, and only mild adverse events such as gastrointestinal complaints have been described previously. The detailed investigation did not reflect any toxicity related to the ingestion of *uva-ursi*. Limited clinical data from small studies suggest that *uva-ursi* effectively prevents UTIs in high-risk patients [174,175]. Using *uva-ursi* as a first-line treatment option is effective in resolving UTI symptoms and reducing antibiotic use. It also leads to favorable effects on resistance rates. Clinical trials.gov identifier (NCT number) of this study is “NCT03151603”.

### 4.3. Vaccinium spp.

Many researchers have suggested that cranberry, *Vaccinium macrocarpon*, is active against UTIs. The plant belongs to the *Ericaceae* family and can be potentially active against *E. coli*, the leading cause of bacteria-mediated UTIs, by reducing bacterial attaching to the bladder’s walls. The bacteria are then more likely to be washed out during urination [176]. It could also inhibit the binding of bacteria to gastrointestinal mucosa [177]. Cranberry contains proanthocyanidins that are stable phenolic compounds and contribute to the anti-adhesion activity against *E. coli*. Therefore, by its consumption, the biofilm formation of Gram-negative and Gram-positive uropathogens such as *S. aureus*, *P. aeruginosa*, *K. pneumonia*, and *Proteus mirabilis* was reduced [178,179].

Cranberry also contains other biologically active constituents like anthocyanidin, catechin, flavanols, myricetin, quercetin, and phenolics, responsible for its activities [180].

Due to the health benefits of cranberry extract, different commercial formulations exist in the market, such as Monoselect Macrocarpon, Anthocran, and Cysticlean. In acute situations, 2–3 capsules/tablets should be taken two to four times per day, and for prevention, one capsule/tablet 2–3 times daily are recommended [181].

Blueberry, Vaccinium myrtillus, has also extensively been used to treat and prevent UTI. Blueberry extracts contain similar constituents as cranberry extracts, with the extracts possessing similar anti-adhesive activities against uropathogenic bacteria. The bacteria are significantly less able to adhere to the bladder walls. Tannins are the most active constituents of blueberry extracts against UTI [182,183].

### 4.4. Sanguiritrin

Sanguiritrin is an original phytomedicine initially developed by scientists at the Institute of Medicinal and Aromatic Plants (VILAR, Russia). It has been made from the upper part of the plants *Macleaya cordata* and *Macleaya microcarpa* and has a different alkaloid composition. It mainly comprises the bisulfates of two benzophenanthridine alkaloids (sanguinarine and chelerythrine), isolated from the stems and leaves of these plants [184,185]. In vitro studies showed that sanguiritrin in concentrations 1–100 µg/mL could effectively suppress the growth of 130 laboratory strains of both Gram-positive and Gram-negative bacteria [40].

Additionally, sanguiritrin was effective against isolated strains resistant to one or more conventional antibiotics. Long-term exposure to this product and multiple passages of the bacterium in its presence did not lead to the development of phytodrug resistance [40]. The primary mechanism of antibacterial action of sanguiritrin is disruption of bacterial cell wall integrity and nucleotide structures and suppression of bacterial nuclease [186]. Treatment with sanguiritrin of *S. aureus* led to single or multiple perforations in the bacterial cell wall and fragmentation [184].

In conclusion, sanguiritrin and other phytodrugs containing benzophenanthridine alkaloids should be considered to treat infections caused by MDR bacteria.

### 4.5. Eucalimin

Eucalimin is a phytodrug constituent of triterpene phenol aldehyde and triterpenoid isolated from foliage and shoots of *Eucalyptus viminalis* Labill [187]. The coupling of phloroglucinol and sesquiterpene constituents is believed to be responsible for this and similar products [188,189]. This product is effective against the growth of Gram-positive bacteria, including clinical isolates of resistant bacteria. However, it was less effective against Gram-negative bacteria and fungi [40]. The antibacterial activities of this product were tested against both Gram-positive and Gram-negative bacteria, with the results showing higher antibacterial activities against Gram-positive bacteria. Gram-positive clinically isolates Staphylococcus (MIC 1.9–31.2 µg/mL), Streptococcus (Streptococcus pneumonia and Streptococcus viridans, MIC 1.9–31.2 µg/mL, Streptococcus faecalis, MIC 0.5–1.9 µg/mL), and Corynebacterium diphtheriae (MIC 3.9–62.5 µg/mL). Gram-positive clinically isolates of *Staphylococcus* (MIC 1.9–31.2 µg/mL), *Streptococcus* (*Streptococcus pneumonia* and *Streptococcus viridans*, MIC 1.9–31.2 µg/mL, *Streptococcus faecalis*, MIC 0.5–1.9 µg/mL), *Corynebacterium diphtheriae* (MIC 3.9–62.5 µg/mL). These results demonstrated the high sensitivity of Gram-positive bacteria to eucalimin. Only Gram-negative bacteria from families of *Acinetobacter* and *Enterobacteriaceae, Serratia* genus, were relatively sensitive to this product (MIC < 125 µg/mL) [40]. Clinical trials suggest that eucalimin is highly efficacious in treating various conditions, including pharyngitis, laryngitis, sinusitis, otitis, colpitis, and cervical erosion.

### 4.6. Scutellaria baicalensis Georgi

The radix of *Scutellaria baicalensis* Georgi (SB), is an important medicinal herb in Japanese and Chinese pharmacopeia. It is traditionally used for inflammatory and infectious diseases, including pathopyretic sores, ulcers, or pustules [190]. The antibacterial functions of SB are due to the active compound baicalein [191]. The mechanism could affect bacterial membrane penetrability, inhibit protein synthesis, and influence SDH, MDH, and DNA topoisomerase I and II to exert antibacterial activities [54,192]. This compound is effective against a wide range of pathogenic bacteria such as *S. aureus*, *Streptococcus mutans, S. pneumonia, E. coli, P. aeruginosa, Salmonella enterica, S. epidermidis*, and *Propionibacterium acnes* [193,194]. A study demonstrated that baicalein exhibited synergistic activities against some extended-spectrum β- lactamases positive *K. pneumonia* strains, especially when combined with cefotaxime, inhibiting CTX-M-1 mRNA expression [195].

The mode of action in combination therapy showed that baicalein with antibiotics caused peptidoglycan and morphological damage, increasing cytoplasmic membrane permeability and protein concentrations and decreased cellular fatty acid and nucleic acid concentrations [196].

The results of another study demonstrated that baicalein could remarkably reverse the ciprofloxacin resistance of MRSA, possibly by inhibiting the NorA efflux pump activity. The inhibition of MRSA pyruvate kinase via baicalein could also lead to a deficiency of ATP, which might further contribute to the antibacterial properties of baicalein against MRSA [197].

Arweiler et al. [198] compared the effects of toothpaste with SB extract (0.5%) with placebo for treatment of gingivitis in 40 participants. The results showed that gingivitis symptoms in the treated group significantly improved compared to the control group.

A study showed that baicalein combined with antibiotics resulted in a higher survival rate in mice severely infected with *S. suis*. At the same time, baicalein can be combined with meropenem against MRSA [199].

Another study showed that baicalein has synergistic antibacterial effects with linezolid in the in vivo model against MRSA biofilms. Furthermore, the inhibitory effects were more pronounced when baicalein was combined with linezolid [200].

### 4.7. Houttuynia Cordata Thunb.

*Houttuynia cordata* Thunb. (HC) is used to treat various diseases such as purulent, suppuration, sores, pustules, and respiratory infections in the Chinese pharmacopeia [40,201]. Houttuynin is the main antibacterial ingredient of HC. This compound and its derivatives are used alone or combined with conventional antibiotics to combat infectious diseases [202,203,204]. HC was found to exhibit anti-biofilm activities against MRSA by inhibiting interleukin-8 (IL-8) and C-C motif chemokine ligand 20 production in human keratinocytes [205]. Kim et al. [206] suggested the HC extract could effectively treat intracellular bacterial infections caused by *Salmonella, Brucella*, *Listeria*, *Bordetella*, and *Helicobacter*. The essential oils of this plant, such as methyl nonyl ketone, bornyl acetate, and β- myrcene, showed antibacterial properties [207]. Another study indicated that the flavonoids of HC had suitable antibacterial activities on *Bacillus subtilis*. The possible antibacterial mechanism is to disintegrate the cell wall, make the cell collapse, and cause content leakage [208].

HC injections were used to treat upper respiratory tract infections and pneumonia, and the results demonstrated that HC injections showed better antipyretic effects than antibiotics in adults [209].

An aqueous extract of HC exhibited virulence reduction activities in *Salmonella typhimurium*-infected BALB/c mice. After a lethal dose of *S. typhimurium*, the mortality rate in the untreated extract group was 100% on the 7th day. Still, at the doses of 25, 50, and 100 μg/mL of extract, groups survived until 11, 17, and 23 days. These data suggest that HC aqueous extract is stable and beneficial in treating bacterial infection, including intracellular replicating pathogens [206].

### 4.8. Berberine

The emergence of antibacterial resistance highlights the need for new therapeutic approaches to ensure the continued effectiveness of conventional antibiotic therapy regimens. Berberine is an alkaloid that has been widely used as an anti-infective agent in traditional medicine. It possesses antibacterial activities against a wide range of microorganisms alone or in combination with antibiotics routinely [210,211,212].

It was shown that the effective concentration of berberine is above 64 µg/mL because of the poor absorption in oral consumption retained in the intestine, reaching a high concentration with distinct benefits for treating intestinal infectious diseases and diarrhea [213]. The encapsulated form of berberine in the yeast cell of *Saccharomyces cerevisiae* has also been shown to have higher stability and bioavailability due to the wall material acting as a barrier, increasing solubility, and sustained release of active material. Berberine-loaded microcapsules had improved MIC against *E. coli* and *S. aureus* compared to berberine alone [214,215]. Berberine has different forms, but the most important ones with proper antibacterial activities are hydrochloride, sulphate, and tannate [216]. Clinical studies showed that a single administration of berberine tannate and its combination with sulfadimidine and neomycin effectively treated acute infective diarrhea in experiments conducted with 55 and 127 children, respectively [217,218]. Compared with standard antibiotic therapy regimens, the recovery of children with acute diarrhea was faster when administering berberine tannate [219].

The hydrochloride form of berberine was found to be more effective than chloramphenicol in 356 and 264 individual cases of patients infected by cholera [35], and the combination of berberine with chloramphenicol and streptomycin exhibited better curative actions on 129 cases of acute diarrheal disorders such as gastroenteritis and bacillary dysentery [220,221].

Apart from the application of berberine in treating gastrointestinal infections, this compound could combat other infections such as urinary tract infections. The in vivo tests were conducted to assess the antibacterial activities of berberine on uropathogenic *E. coli* strains. *Galleria mellonella* as an infection model was used to confirm berberine’s ability to reduce bacterial adhesion and invasion proprieties of *E. coli* on human bladder cells. The results indicated that increasing the surviving larvae infected with pathogens reduced circulating uropathogenic *E. coli* strains in larvae hemolymph [210].

In another study, the efficacy and safety of berberine hydrochloride, amoxicillin, and rabeprazole triple therapy versus bismuth-containing quadruple therapy (amoxicillin, clarithromycin, rabeprazole, and bismuth) in the first eradication treatment of *Helicobacter pylori* were assessed. It is hypothesized that berberine hydrochloride, amoxicillin, and rabeprazole triple therapy are non-inferior to bismuth-containing quadruple therapy. Clinical trials.gov identifier (NCT number) of this study is “NCT04697186”.

### 4.9. Mastic

Mastic is a semi-translucent pastel yellow-to-white resin obtained from *Pistacia lentiscus*. It comprises polymer *cis*-1,4-poly-β- myrcene, triterpenoids, sterols, and simple phenolics [216]. It also contains about 2% of an essential oil composed mainly ofα-pinene, producing weak antibacterial activities against *H. pylori*. However, α-terpineol and (E)-methyl isoeugenol, minor constituents of mastic essential oil, showed significant inhibitory effects against *H. pylori* strains [222,223]. In addition, mastic extract containing arabinogalactan proteins inhibited neutrophil activation and growth of *H. pylori*, suggesting its role in eliminating helicobacter infections [224].

This compound has been used in various dietary supplements and traditional medicines in different dosage forms such as capsules, oil extracts, and tablets for the protection and treatment of gastrointestinal health, gastric ulcers, healing peptic, relief of gastric and intestinal inflammation and also used as a natural treatment for *H. pylori* infections. In recent decades, several clinical trials were performed to evaluate the effect of mastic in *H. pylori* eradication. A double-blind clinical trial was carried out with 38 patients, and mastic was given orally (1 g daily). After two weeks of consumption, the results showed that relieving symptoms and healing duodenal ulcers were significantly more effective than placebo [225]. Additionally, administering 350 mg of mastic three times a day for 14 days to 52 patients demonstrated an effect on eradicating *H. pylori* [226]. The other study was performed with five *H. pylori*-infected patients and three healthy controls who received 1 g of mastic daily for two months. The results showed the inhibitory effects on *H. pylori* neutrophil-activating protein involved in *H. pylori*-related gastric pathologies [227].

### 4.10. GutGard

GutGard encapsulated standardized root extract of *Glycyrrhiza glabra* contains flavonoids (more than 10% *w*/*w*), mainly glabridin and glabrol, along with saponins (glycyrrhizin), and phenylpropanoids (eicosanyl caffeate and docosyl caffeate). GutGard exhibited in vitro anti-*H. Pylori* activities with MIC values ranging from 32 to 100 μg/mL. In addition, glabridin has MIC values of 12.5 μg/mL against various strains, including clarithromycin and amoxicillin-resistant [228,229]. Moreover, an aqueous extract of *G. glabra* significantly inhibited the adhesion of *H. pylori* to human stomach tissue. Its anti-adhesive properties were related to the polysaccharides, which did not have direct toxic effects against *H. pylori* [230].

This product could be used as an herbal supplement to combat *H. pylori* infections and their symptoms. It is claimed that GutGard can reduce abdominal fullness and pain, belching, bloating, dyspepsia, nausea, and *H. pylori* loading. It should be noted that different regulatory agencies such as the Committee on Herbal Medicinal Products (HMPC) of the European Medicines Agency (EMA) approve *G. glabra* root for the relief of digestive symptoms, such as burning sensation and dyspepsia. A randomized, double-blind placebo-controlled study with 107 *H. pylori*-infected patients received orally 150 mg of GutGard once daily for 60 days. The results revealed a significant decrease in the *H. pylori* gastric load compared to a placebo group, with the product safe and well-tolerated [231]. Other clinical experiments carried out with deglycyrrhizinised *G. glabra* extract clearly showed the product’s effectiveness in treating and preventing gastric ulcers [232,233].

### 4.11. Listerine

Listerine Antiseptic Mouthwash is one of the highest-selling products in the United States and other markets. This product comprises essential oils from *Eucalyptus* spp., *Gaultheria* spp., *Mentha × piperita,* and *Thymus vulgaris*. It contains a mixture of their main constituents, eucalyptol and thymol, responsible for the antimicrobial activities, menthol and methyl salicylate, local anesthetic, and cleaning agent, respectively [234]. The long-term plaque- and gingivitis-reducing properties of this product have been confirmed in several clinical trials. For example, in a study by Sharma et al. [235], Listerine was remarkably more effective in controlling gingivitis and plaque formation than the control group. In another study, both Listerine and chlorhexidine mouthwashes significantly reduced plaque formation and gingivitis compared to the control [236]. The superiority of rinsing with Listerine in reducing plaque and gingivitis was demonstrated compared to rinsing with cetylpyridinium chloride or hydroalcoholic (control) solutions [237]. The ability of this product to reduce gingivitis- and plaque-reducing was also be found in other similar studies [238,239].

### 4.12. Parodontax

Parodontax (GlaxoSmithKline, Brentford, UK) is a toothpaste (also available as a mouthwash) composed of the extracts of *Commiphora myrrha*, *Echinacea purpurea*, *Krameria triandra,* and *Matricaria recutita* together with the essential oils from *Mentha arvensis*, *M. x piperita,* and *Salvia officinalis*. *M. recutita* and *S. officinalis* have been used traditionally to treat minor ulcers and inflammations of the mouth and throat [216]. The in vitro antimicrobial activities of Parodontax and its herbal components, *S. officinalis* essential oil, were tested. The results showed a significant effect against *C. albicans* with MIC values ranging from 16 to 2780 μg/mL. The essential oil of another herbal ingredient, *M. arvensis*, also inhibited the growth of *Prophyromonas gingivalis* in both planktonic and biofilm forms with relatively high MBC values ranging from 6 and 26 mg/mL, respectively [240,241].

In a clinical study conducted with eight adult volunteers, this product significantly reduced dental plaque regrowth after four days and plaque vitality over a period of 24 h compared to a control group [242]. The efficacy of Parodontax on the reduction of plaque and gingivitis was also demonstrated in a randomized, double-blind controlled study, a 28-day trial performed with 48 volunteers with established gingivitis [243]. In another study, the product showed antimicrobial activities against oral biofilms of different compositions (for example, of *Actinomyces naeslundii* and *Streptococcus oralis*) and maturational status [244].

### 4.13. Myrtol

Myrtol, a herbal medical product, is sold in gelatin capsule form and is recommended for reducing the risk of acute exacerbations from chronic bronchitis. A distillation procedure obtains it from various essential oils such as *Citrus limon*, *Citrus sinensis*, *E. globulus*, and *M. communis*. The monoterpenes D-limonene, eucalyptol, and α- pinene are the major biological active substances in this product [216]. The product shows a wide range of biological properties such as antimicrobial, mucociliary clearance, anti-inflammatory, and antioxidant [245]. HMPC approves *E. globulus* leaves and essential oil as a traditional herbal medicinal product used to relieve cough associated with cold. The product has proven to effectively treat acute and chronic respiratory infections in both adults and children in several clinical trials [246,247,248]. Myrtol was compared to prescription drugs cefuroxime and ambroxol in the double-blind, placebo-controlled study with 681 patients suffering acute bronchitis. The results showed it was superior to the control group and comparable to the prescribed drugs for faster and more complete recovery [248]. 

### 4.14. Tea Tree Oil

One of the best-known examples of topical herbal antiseptics is tea tree oil (TTO). It is composed of essential oil from *M. alternifolia* leaves. It is commonly sold in a pure form and is part of cosmetic products, including antiseptic wipes, balms, body lotions, creams, deodorants, gels, shampoos, and ointments [216]. HMPC approved TTO to treat small boils (furuncles and mild acne), minor superficial wounds, and insect bites to relieve itching and irritation. Several clinical trials demonstrated the antimicrobial properties of TTO against various important bacterial skin infections, dermatophytes, and dandruff [249,250,251]. Two clinical trials confirmed TTO activity against acne. 124 patients were randomly divided into groups in the first trial and treated with 5% TTO and 5% benzoyl peroxide. Both treatments had significant effects on reducing the number of lesions [249]. In another clinical trial with 60 patients, the effect of 5% TTO gel was compared with the placebo group. The TTO group was significantly more effective in reducing lesion count after 45 days, whereas the adverse effects were tolerable and similar to those of the placebo [250]. TTO 10% cream, 5% body wash, chlorhexidine 4%, and silver sulfadiazine 1% were used for MRSA decolonization of superficial skin sites and skin lesions. This study indicated that the TTO formulations were more effective than the drug control groups [251]. In a randomized controlled trial with 391 patients, the 5% TTO body wash was able to prevent MRSA colonization with respect to the standard non-medicated body wash. However, there were no significant differences between the two groups; TTO was evaluated as safe and well-tolerated [252]. In a randomized, double-blind clinical trial with 104 patients, the efficacy of 10% w/w TTO cream in the treatment of athlete’s foot was compared with 1% tolnaftate and placebo creams.TTO cream reduced the symptomatology of the athlete’s foot as effectively as tolnaftate [253].

## 5. Conclusions

New resistant bacterial strains have led to a paradigm shift from conventional antibiotic therapy to alternative approaches. Plants represent an attractive source of antimicrobial agents with therapeutic potential as alternatives or potentiators of antibiotics. The high chemical diversity of active constituents found in plants makes them a potential source of antibacterial agents. To this end, identifying new and valuable antibacterials from plants and testing their antibacterial properties are crucial and need to be carefully studied. Two reasons may play pivotal roles among several reasons to employ herbal-derived compounds for combating bacterial infections. First, these compounds could provide other mechanisms of antibacterial action with respect to conventional antibiotics. Second, the use of unique traditional knowledge of herbal medicine provides the excellent potential to generate biocompatible, cost-effective, and promising solutions and will hasten the discovery of new antibacterial agents. Therefore, in silico, in vitro, and in vivo tests and models have been developed to evaluate phytomedicines’ antibacterial activities, mechanism of action, and biological fate. Understanding their mechanisms of action helps us choose the appropriate phytomedicine for a specific situation and particular microorganism.

Despite the rapid increase in publications on antibacterial plant compounds, few plant-derived drugs are still in clinical use. This might be because phytomedicines often require complex combinational effects between their bioactive compound to synergize the activities of their components. Therefore, examining combinations of plant compounds with conventional antibiotics may be a more fruitful line of research.

## Figures and Tables

**Table 2:** Examples of t herbal-derived products as antibacterial agents in clinical trials or markets.

Compound or Product	Sources or Ingredient	Indications
Concentrated herbal extract granules TRA	Traditional Chinese Medicine	Urinary tract infections
*Uva ursi* extract	*Uva ursi*	Urinary tract infections
Monoselect Macrocarpon	Vaccinium spp.	Urinary tract infections
Anthocran	Vaccinium spp.	Urinary tract infections
Cysticlean	Vaccinium spp.	Urinary tract infections
UVA-E	*Arctostaphylos uva-ursi*, *Taraxacum officinale*	Urinary tract infections
Pylorin	polyherbal formulation	*Helicobacter pylori* Infection
Sanguiritrin	*Macleaya cordata* and *Macleaya* *microcarpa*	Acute intestinal infections and wound infections
Eucalimin	Consisted of triterpene phenol aldehyde and triterpenoid that isolated from foliage and shoots of *Eucalyptus Viminalis* Labill	Pharyngitis, laryngitis, and sinusitis
*Scutellaria baicalensis* Georgi	*Scutellaria baicalensis* Georgi	Pathopyretic sores, ulcers or pustules
Houttuynia cordata Thunb		Pseudorabies herpesvirus
Berberine	* Berberis vulgaris *	Gastrointestinal infections
Mastic	*Pistacia lentiscus* resin	*H. pylori* Infection
GutGard	*Glycyrrhiza glabra* extract	*H. pylori* Infection
Listerine	eucalyptol, menthol, methyl salicylate, and thymol	Oral infections
Parodontax	*Commiphora myrrha*, *Echinacea purpurea*, *Krameria triandra*, and *Matricaria recutita* extracts; *Mentha arvensis*, *M. x Piperita* and *Salvia officinalis* essential oils	Oral infections
Myrtol	*Citrus limon*, *Citrus sinensis*, *Eucalyptus globulus*, and *Myrtus communis* essential oils	Chronic and acute bronchitis
Tea tree oil	TTO, *Melaleuca alternifolia* essential oil	Mild to moderate acne
